# Environmental heat stress on maternal physiology and fetal blood flow in pregnant subsistence farmers in The Gambia, west Africa: an observational cohort study

**DOI:** 10.1016/S2542-5196(22)00242-X

**Published:** 2022-12-07

**Authors:** Ana Bonell, Bakary Sonko, Jainaba Badjie, Tida Samateh, Tida Saidy, Fatou Sosseh, Yahya Sallah, Kebba Bajo, Kris A Murray, Jane Hirst, Ana Vicedo-Cabrera, Andrew M Prentice, Neil S Maxwell, Andy Haines

**Affiliations:** a Medical Research Unit The Gambia at London School of Hygiene & Tropical Medicine, Fajara, The Gambia; b Centre on Climate Change and Planetary Health, London School of Hygiene & Tropical Medicine, London, UK; c MRC Centre for Global Infectious Disease Analysis, School of Public Health, Imperial College London, London, UK; d Nuffield Department of Women's and Reproductive Health and the George Institute for Global Health, University of Oxford, Oxford, UK; e Institute of Social and Preventive Medicine, University of Bern, Bern, Switzerland; f Oeschger Center for Climate Change Research, University of Bern, Bern, Switzerland; g Environmental Extremes Laboratory, University of Brighton, Eastbourne, UK

## Abstract

**Background:**

Anthropogenic climate change has caused extreme temperatures worldwide, with data showing that sub-Saharan Africa is especially vulnerable to these changes. In sub-Saharan Africa, women comprise 50% of the agricultural workforce, often working throughout pregnancy despite heat exposure increasing the risk of adverse birth outcomes. In this study, we aimed to improve understanding of the pathophysiological mechanisms responsible for the adverse health outcomes resulting from environmental heat stress in pregnant subsistence farmers. We also aimed to provide data to establish whether environmental heat stress also has physiological effects on the fetus.

**Methods:**

We conducted an observational cohort study in West Kiang, The Gambia, at the field station for the Medical Research Council Unit The Gambia at London School of Hygiene & Tropical Medicine (named the MRC Keneba field station). Pregnant women who were aged 16 years or older and who were at <36 weeks’ gestation of any gravida or parity were invited to participate in the study. Participants were eligible if they were involved in agricultural or related manual daily tasks of living. Participants were ineligible if they refused to provide consent, had multiple pregnancies (eg, if they had twins), were acutely unwell, or were diagnosed with pre-eclampsia or eclampsia. Heat stress was measured by wet bulb globe temperature (WBGT) and by using the universal thermal climate index (UTCI), and maternal heat strain was directly measured by modified physiological strain index calculated from heart rate and skin temperature. Outcome measures of fetal heart rate (FHR) and fetal strain (defined as a FHR >160 beats per min [bpm] or <115 bpm, or increase in umbilical artery resistance index) were measured at rest and during the working period. Multivariable repeated measure models (linear regression for FHR, and logistic regression for fetal strain) were used to evaluate the association of heat stress and heat strain with acute fetal strain.

**Findings:**

Between Aug 26, 2019, and March 27, 2020, 92 eligible participants were recruited to the study. Extreme heat exposure was frequent, with average exposures of WBGT of 27·2°C (SD 3·6°C) and UTCI equivalent temperature of 34·0°C (SD 3·7°C). The total effect of UTCI on fetal strain resulted in an odds ratio (OR) of 1·17 (95% CI 1·09–1·29; p<0·0001), with an adjusted direct effect of OR of 1·12 (1·03–1·21; p=0·010) with each 1°C increase in UTCI. The adjusted OR of maternal heat strain on fetal strain was 1·20 (1·01–1·43; p=0·038), using the UTCI model, with each unit increase.

**Interpretation:**

Data from our study show that decreasing maternal exposure to heat stress and heat strain is likely to reduce fetal strain, with the potential to reduce adverse birth outcomes. Further work that explores the association between heat stress and pregnancy outcomes in a variety of settings and populations is urgently needed to develop effective interventions.

**Funding:**

The Wellcome Trust.

## Introduction

Anthropogenic climate change has increased global annual temperatures by approximately 1·2°C compared with pre-industrial temperatures.^1^ Without major decreases in greenhouse gas emissions, this trend will continue, with potentially catastrophic impacts on human health.^2^ Large epidemiological studies have shown that there is an increased risk of adverse birth outcomes in mothers who are exposed to high ambient temperatures, including premature birth, low birthweight, stillbirth, pre-eclampsia, and gestational diabetes.^3^, ^4^, ^5^

Although global temperatures are rising, some regions will be affected by the impacts of climate change more than others.^6^, ^7^ Populations in west Africa are highly susceptible to the impacts of climate change and will be increasingly exposed to extreme heat.^8^ However, data for the impact of heat on pregnancy outcomes in Africa are sparse.^9^ The at-risk population is large, with around 200 million women living in west Africa alone, with this number estimated to reach 400 million by the year 2050.

**Research in context**

**Evidence before this study**

We searched Embase for articles published between 1947 and Sept 9, 2022, using the following search terms: (“heat” or “heat stress” or “heat wave”) AND (“pregnancy” and “pregnancy complication” or “low birth weight” or “prematurity” or “stillbirth”) AND (“physiology”). Although there were many studies showing a clear association between maternal exposure to high ambient temperature and poor birth outcomes, the physiological mechanisms have not been defined. Hypothesised mechanisms are based on either animal, fever, or heat chamber studies. To the best of our knowledge, there is no evidence of the pathophysiological pathways between heat stress and maternal and fetal health outcomes from real-world field studies.

**Added value of this study**

In this study, we directly measured the heat stress exposure of pregnant subsistence farmers, showing that they commonly experience extreme levels of heat during a normal working day. This heat stress was significantly associated with fetal strain even when controlling for maternal heat strain. Maternal heat strain was also associated with fetal strain.

**Implications of all the available evidence**

Our findings show that in The Gambia, pregnant subsistence farmers are frequently exposed to extreme heat stress, leading to maternal heat strain. This heat strain also has a detectible impact on the fetus. The development and evaluation of interventions to reduce maternal heat strain and the resulting effects on the fetus are therefore urgently needed.

In The Gambia, as in many similar countries, women make up at least 50% of the agricultural workforce.^10^ Outdoor workers, who lack the choice to avoid heat exposure, are at an increased risk of heat strain, although data from Africa are scarce.^11^ The growing risk of climate change to both livelihoods and health, particularly for women, is a neglected research topic that needs further work to identify problems and determine actionable solutions.

During pregnancy, the effects of heat stress (defined as the environmental exposure of heat, humidity, solar radiation, and wind speed, while heat strain is defined as the physiological response to that stress) are exacerbated by an increased physiological demand from the fetus and placenta. The physiological mechanisms behind adverse birth outcomes are not clearly defined. Hypotheses from animal and human fever studies speculate that raised core temperature, dehydration, reduced uterine blood flow, inflammatory changes, and heat shock proteins could be involved.^12^, ^13^, ^14^

Although there is emerging evidence to show that temperature control in pregnancy is conserved,^15^ there remain concerns with regard to prolonged ambient exposure and extreme heat.^16^ To develop targeted interventions to reduce the risk of adverse birth outcomes, a more complete understanding of the physiological mechanisms involved is required.^5^

This observational cohort study of pregnant, subsistence-farming women in The Gambia aimed to assess environmental heat stress on maternal and fetal physiology, in a region and population where high levels of heat stress are often observed. We aimed to assess the effects of working in the heat and to examine whether these populations are at risk of heat strain. In addition, this study also aimed to establish whether there are also physiological effects on the fetus.

## Methods

### Study design and participants

This observational cohort study was set in West Kiang, a rural area that is approximately 150 km from the capital of The Gambia, where the field station for the Medical Research Council Unit The Gambia at London School of Hygiene & Tropical Medicine is located (named the MRC Keneba field station). West Kiang is populated by 34 villages, with populations ranging from 200 to 2000 people. Antenatal care is provided free of charge at the MRC Keneba field station, where women attend antenatal appointments every month, are routinely supplemented with iron and folic acid, and around 30% of whom attend for delivery.^17^ At the time of the study, most villages did not have access to gridded electricity or piped running water.

Pregnant women who were aged 16 years or older and who were at fewer than 36 weeks’ gestation of any gravida or parity were invited to participate in the study. Participants were eligible if they were involved in agricultural or related manual daily tasks of living (including water collection, gardening, rice planting, harvesting, and washing clothes). Participants were ineligible if they refused to provide consent, had multiple pregnancies (eg, if they were pregnant with twins), were acutely unwell, or were diagnosed with pre-eclampsia or eclampsia (appendix p 3). All participants gave written informed consent to participate in the study**.**

**Figure 1:**
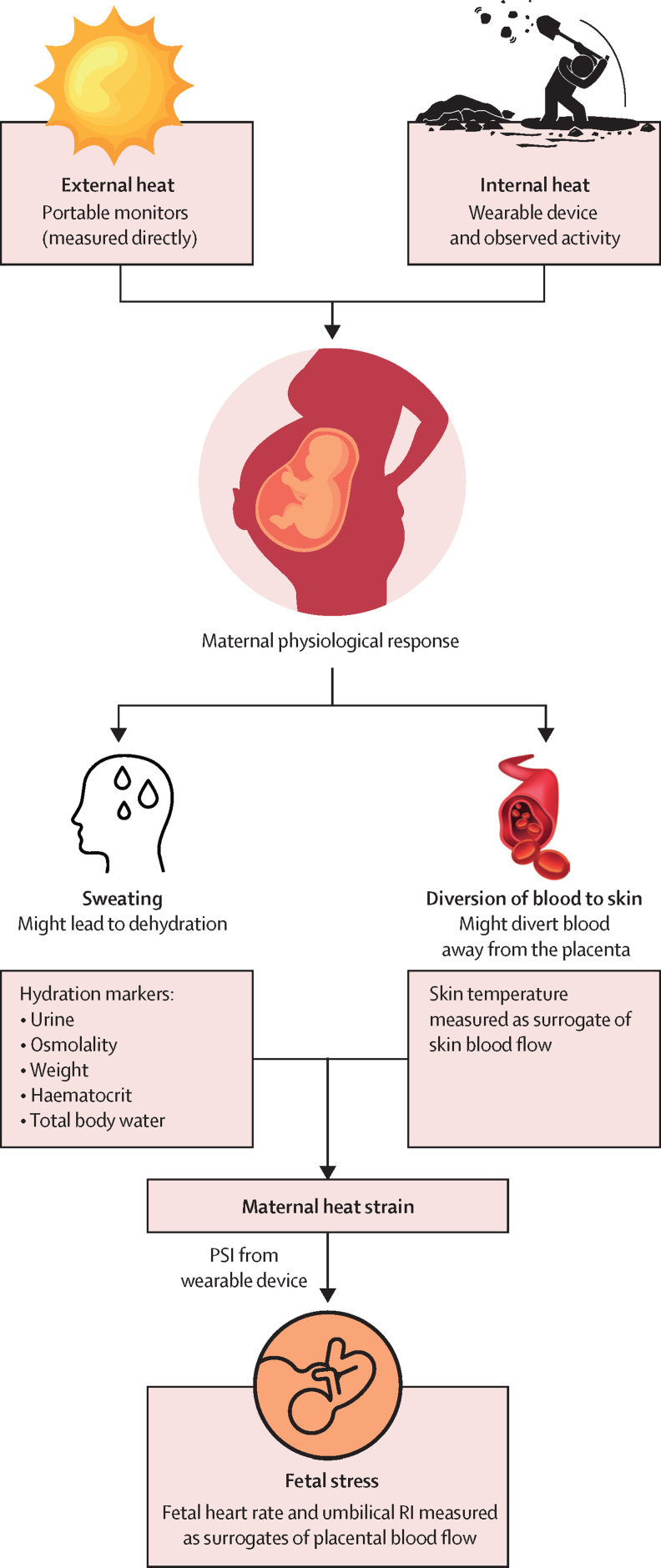
Maternal physiological responses to thermal heat stress Measurements taken at each field visit are shown in grey. PSI=physiological strain index. RI=resistance index of umbilical artery.

The study was approved by the Gambian Government and the Medical Research Council Unit The Gambia joint ethics committee and the London School of Hygiene & Tropical Medicine ethics advisory board in accordance with the Declaration of Helsinki (2013). The published study protocol gives full details of the methods.^18^ Figure 1 provides a summary of the physiological pathways investigated and relevant measurements taken.

### Procedures and outcomes

Baseline data for previous medical conditions, obstetric history, and current pregnancy were collected for this study. Gestational age was ascertained through last menstrual period (if known) or biparietal diameter, which was measured by ultrasound scan. An ultrasound scan at each visit was conducted to measure fetal growth and to identify anomalies. The study team were scheduled to attend field visits with participants every 2 months until the time of delivery.

During field visits, participants were encouraged to carry out their usual daily tasks. Hourly air temperature, relative humidity, black globe temperature, wet bulb globe temperature (WBGT), and wet bulb and dew point data were measured throughout the participants’ outdoor working shift using the HT200: Heat Stress WBGT Meter (Extech, Nashua, NH, USA). Wind speed was measured using a portable anemometer (Extech AN100 thermo-anemometer, Nashua, NH, USA). From these measurements, the universal thermal climate index (UTCI) was calculated using the alfrisci/rBiometeo package.^19^ Both average conditions and peak conditions were calculated for WBGT, UTCI, air temperature, and relative humidity for each participant during each visit. WBGT is widely used in both occupational health regulations and in research. Our decision to measure WBGT was to aid the comparability and potential generalisability of our findings. WBGT is a continuous measure but has been divided into five categories, from no risk (0) of heat illness in occupational settings to extreme risk (5) of heat illness in occupational settings. The UTCI, a more recent heat stress index, is based on a physiological model and is potentially better than WBGT at expressing biothermal conditions. The UTCI is divided into ten categories that range from extreme cold stress to extreme heat stress.^20^ To the best of our knowledge, there are no modifications that have been specifically developed for pregnancy in any of the heat indexes and therefore both were evaluated.

Participants were fitted with an Equivital LifeMonitor (Cambridge, UK), which is a wearable device with inbuilt fabric sensors. This device continuously recorded maternal heart rate, skin temperature, and triaxis accelerometer data.

Duplicate tympanic temperature measurements from both membranes were taken using a Braun ThermoScan 7 tympanic thermometer (Braun, Kronberg, Germany) and the highest measurement was recorded at each timepoint.^21^, ^22^

The physiological strain index (PSI) has been widely accepted as an accurate measurement of heat strain.^23^ This model has been validated for men and women, but there are no data for its use during pregnancy. PSI requires continuous core temperature measurements, but these were unavailable owing to an absence of data for the safety of telemetry pills in pregnancy and therefore we used continuous chest skin temperature measurements to give a modified PSI (PSI_MOD_), using the following equation:^23^, ^24^

$${\text{PSI}}_{\text{MOD}}=5*(\left({\text{T}}_{\text{SKT}}-{\text{T}}_{\text{TYMP0}}\right)/\left(39.5-{\text{T}}_{\text{TYMP0}}\right)+5*(\left({\text{HR}}_{\text{t}}-{\text{HR}}_{\text{0}}\right)/\left(180-{\text{HR}}_{\text{0}}\right)$$

wherein skin temperature at time t (T_SKT_), tympanic temperature at baseline (T_TYMP0_), heart rate at time t (HR_t_), and heart rate at rest (HR_0_) are represented. Use of triaxial accelerometer data in pregnancy to estimate energy expenditure is problematic.^25^ We increased the robustness of our estimates by calculating the physical activity energy expenditure (PAEE) with two methods. First, PAEE was calculated from the LifeMonitor data using the following validated equation (method A), with the standard 6 min walk test used to calibrate the device:^26^

$$\text{PAEE}=\left(\text{5}*\text{HRaS}\right)+\left(\left(023*\text{HRaS}\right)*{\text{HRaS}}_{\text{3min}}\right)+\left(22*{\text{HRaS}}_{\text{3min}}\right)+9\cdot 2*\log\left(\text{RMSDD}\right)-\left(2.6*\text{SHR}\right)-82)$$

wherein heart rate above sleeping or resting heart rate (HRaS), average heart rate above resting heart rate at 3 min into the calibration test (HRaS_3min_), root mean square of successive difference of inter-beat intervals during calibration test (RMSSD), and sleeping or resting heart rate (SHR) are represented.

Second, energy expenditure was determined on the basis of direct activity observations of each participant matched to historic indirect calorimetry data for energy expenditure by task in pregnant women in West Kiang, determined by Lawrence and colleagues (method B),^27^ which was given in metabolic equivalents (METs). Where estimates from the LifeMonitor were outside the expected range (2–15 METs), historical data were used.

Measures of hydration, body composition, haematology, and biochemistry were measured at the beginning and end of each field visit. The TANITA BC-418MA segmental body composition analyser uses bioimpedance to measure free fat mass, fat percentage, total body water, and basal metabolic rate.

Maternal symptoms of heat illness were collected at the midpoint and endpoint of field visits. According to Centers for Disease Control and Prevention guidance and other published literature,^11^, ^28^ self-reported symptoms included nausea, vomiting, headache, dizziness, weakness, muscle ache, fatigue, and dry mouth.

Ultrasound for biometry, fetal heart rate (FHR), placental position, and assessment of liquor volume was conducted before every field visit by a trained midwife. Umbilical artery doppler is an established non-invasive tool for measuring placental blood flow and gives information on the maternal–fetal circulation.^29^ The UmbiFlow device is a portable, low-cost, continuous wave doppler that was developed in South Africa and is validated for use in measuring umbilical artery blood flow. In eligible participants (those at ≥28 weeks’ gestation), a baseline recording of the resistance index (defined as [systolic velocity minus diastolic velocity] divided by systolic velocity) was recorded at this time. FHR and resistance index measures that were taken at this time were taken as the baseline measurements.

During field visits, FHR and (if eligible) the umbilical artery resistance index were recorded at the midpoint and the endpoint of the working shift. The umbilical artery resistance index was recorded twice at each timepoint and assessed for quality. An outcome of fetal strain was defined as a FHR greater than or equal to 160 beats per min (bpm), less than or equal to 115 bpm for 5 min, or an increase in umbilical artery risk category from baseline, based on the definition of fetal distress and in keeping with clinical practice and current evidence.^30^ These measurements were used as surrogate markers for impact on maternal–fetal circulation.

Data for gestational age, birthweight (within 72 h of delivery), infant sex, mode of delivery, place, level of training of the attendant at delivery, and infant or maternal mortality were collected.

The primary outcome of the study was presence of fetal strain, which was assessed in all participants at each field visit. The theoretical framework of this work has been described previously.^18^ As fetal heat strain has never been defined previously and would be extremely challenging to measure, especially in the field, we use surrogate measures (fetal heart rate and umbilical artery resistance index) to determine the effect of heat stress on blood flow to the fetus, defined below as fetal strain. This study was not powered to assess the effect of heat strain on birth outcomes.

### Statistical analysis

The initial sample size estimation in our study protocol was based on 35% of workers being exposed to heat stress (WBGT >24·8°C).^11^, ^18^ An updated sample size calculation that included an increased exposure level and that assumed an unexposed incidence risk of fetal strain to be 5% estimated that a sample size of 74 would be needed to detect an exposure incidence risk of 30%, with an α of 0·05 and a power of 80%.

All analyses were conducted in R, version 4.1.0. Normally distributed continuous variables are presented as a mean with SD, non-parametric data as median and IQR, and categorical variables as counts. Heat stress exposure variables were analysed as continuous data. Outcome measures of fetal strain were analysed by both FHR as a continuous variable and fetal strain as a binary variable as defined in the Procedures and outcomes section. Initial data exploration assessed changes in mean temperature and heart rate from baseline to working state using Wilcoxon signed-rank tests. The correlation between multiple similar variables (eg, WBGT, UTCI, and air temperature) were evaluated using Pearson's correlation. Univariable analysis of maternal heat strain (PSI_MOD_), fetal strain by FHR (continuous variable and linear regression), and fetal strain (binary variable and logistic regression) were conducted to explore risk factors. Final datasets in multivariable analyses were complete.

The association between heat stress and maternal heat strain (by PSI_MOD_) was explored using linear and non-linear models.^31^ Non-linear models with natural and logarithmic splines with knots placed at the 50th and 90th centiles were evaluated, in keeping with other studies on the association between temperature and health outcomes. The linear model had the lowest Akaike information criterion and was used in the subsequent repeated measures multivariable models.

To explore the effect of heat stress and maternal heat strain on the fetus, we used two multivariable repeated measures models with FHR (model A, linear regression) and fetal strain (model B, logistic regression) as outcomes. All variables were decided a priori on the basis of biological plausibility and directed acyclic graphs (appendix pp 13–14). Model 1 shows the total effect of heat stress on fetal strain or FHR:

$${\text{Y}}_{\text{ij}}∼\beta_{\text{0}}+\beta_{\text{1}}*\text{Heat}\;{\text{stress}}_{\text{ij}}$$

wherein fetal strain or fetal heart rate for individual *i* at time *j* (Y) and heat stress exposure for individual *i* at time *j* are represented. Model 1 gives the estimate of effect (model A) and the odds ratio (OR; model B) for each 1°C increase in heat stress on fetal strain to give the total effect of heat stress.

Model 2 gives the direct effect of heat stress on fetal strain while controlling for maternal heat strain:

$${\text{Y}}_{\text{ij}}∼\beta_{\text{0}}+\beta_{\text{1}}*\text{Heat}\;{\text{stress}}_{\text{ij}}+\beta_{\text{2}}*\text{Heat}\;{\text{strain}}_{\text{ij}}$$

wherein FHR or fetal strain for individual *i* at time *j* (Y), UTCI or WBGT for individual *i* at time *j* (heat stress), PSI_MOD_ of individual *i* at time *j* (heat strain), estimation of cardiovascular fitness determined by distance travelled in standardised 6 min walk test for individual *i* at time *j* (fitness), and measurement of body fat (as BMI is not useful in pregnancy) for individual *i* at time *j* (% fat mass) are represented. Model 2 gives the estimate of effect (model A) and the OR (model B) for each 1°C increase in heat stress on fetal strain, controlling for maternal heat strain. This model estimates the effects of heat stress on fetal strain due to other mechanisms outside of maternal heat strain.

Model 3 gives the direct effect of maternal heat strain on fetal strain while controlling for heat stress, cardiac fitness, percentage of fat mass, and gestational age:

$${\text{Y}}_{\text{ij}}∼\beta_{\text{0}}+\beta_{\text{1}}*\text{Heat}\;{\text{stress}}_{\text{ij}}+\beta_{\text{2}}*\text{Heat}\;{\text{strain}}_{\text{ij}}+\beta_{\text{3}}*{\text{Fitness}}_{\text{ij}}+\beta_{\text{4}}*\%{\text{fat mass}}_{\text{ij}}+\beta_{\text{5}}*{\text{Gestational age}}_{\text{ij}}$$

wherein FHR or fetal strain for individual *i* at time *j* (Y), UTCI or WBGT for individual *i* at time *j* (heat stress), PSI_MOD_ of individual *i* at time *j* (heat strain), estimation of cardiovascular fitness determined by distance travelled in standardised 6 min walk test for individual *i* at time *j* (fitness), and measurement of body fat (as BMI is not useful in pregnancy) for individual *i* at time *j* (% fat mass) are represented. Full details of all models and analysis code can be found in the appendix (pp 19–22). The final models were assessed for violation of model assumptions by assessing the linearity of residuals, homoscedasticity by Levene's Test, and the normal distribution of residuals.

### Role of the funding source

The funder of the study had no role in study design, data collection, data analysis, data interpretation, or writing of the report.

## Results

Study recruitment occurred from Aug 26, 2019, to March 27, 2020, with data collection completed on Dec 17, 2020. All 92 participants attended at least one field visit, with 30 (33%) attending a second field visit (appendix p 3). The demographics, physical characteristics, and birth outcomes of all participants are shown in table 1.

**Table 1:** Demographics, physical characteristics, and birth outcomes of participants

		**Participants (n=92)**
Age, years		27·7 (23·7–35·8)
Schooling, years in education		5 (4–8)
Ethnicity		
	Mandinka	85 (92%)
	Wolof	6 (7%)
	Fula	1 (1%)
Occupation		
	Farmer	74 (80%)
	Other	18 (20%)
Marital status		
	Single	2 (2%)
	Married	89 (97%)
	Widowed	1 (1%)
Gravida		4 (2–7)
Parity		3 (1–5·25)
Gestational age at visit, weeks		28·5 (23·6–32·9)
Height, cm		162·9 (5·6)
Weight		
	Median, kg	61·9 (55·8–97·3)
	BMI, kg/m^2^	23·0 (21·3–25·9)
	MUAC, cm	27·0 (25·1–29·7)
Percentage fat mass, %		28·7% (25·8–33·8)
Birth outcome		
	Normal birth	53/91 (58%)*
	SGA	24/91 (26%)*
	Preterm	12/92 (13%)
	Low birthweight	12/91 (13%)*
	Stillbirth or intrapartum death	3/92 (3%)

Data are median (IQR), n (%), or mean (SD). MUAC=mid-upper arm circumference. SGA=small for gestational age.

* Data are missing for one participant.

During the study, the COVID-19 pandemic occurred. The Gambia entered lockdown on March 27, 2020, and all study activities stopped, as mandated by the Gambian Government and the MRC Keneba field station. The pandemic affected the study in several ways. First, intending to have a full year of directly measured meteorological exposure, we only have 7 months. Second, our initial sample size calculations required 99 participants. By the end of the study period we had data for 92 participants. Third, we expected to have most participants attend at least two field visits, with some attending up to four field visits. Instead, most participants attended only one field visit, and fewer than half of participants attended two field visits. Last, during the lockdown, retrospective birth outcomes were collected from the antenatal card. However, if data were not recorded they were coded as missing.

**Figure 2:**
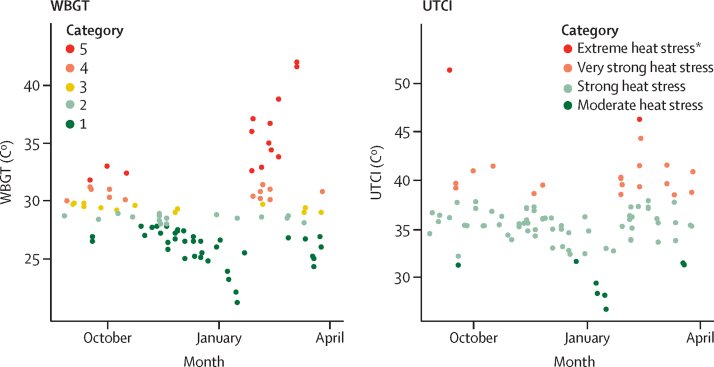
Maximum measured heat stress exposures of participants during a working shift WBGT is coded by risk category for heat illness, whereby 1=no risk, 2=low risk, 3=moderate risk, 4=high risk, and 5=extreme risk. WBGT=wet bulb globe temperature. UTCI=universal thermal climate index. *Extreme heat stress was defined as the scenario in which temperatures exceeded 46°C.

Field visits occurred during the rainy season (August to October) and the first half of the dry season (November to March). Throughout the study period, mean air temperature was 33·5°C  (SD  3·8°C, range 22·1–45·4°C) and humidity varied substantially (mean 28·1 % (SD  23·4%, range 0–87·7%). Mean WBGT was 27·2°C (SD 3·6°C), with a maximum value of 42·0°C. In 38 (31%) of 122 field visits, the WBGT category was four or above. Mean UTCI was 34·0°C (SD 3·7°C), with maximum values reaching 51·3°C. 25 (20%) of 122 field visits showed that pregnant women were exposed to very strong heat stress and above (ie, >46°C; figure 2). Comparisons between measured values and monthly weather station results are presented in the appendix (p 4). However, in this study, 80% of field visits occurred under heat stress conditions. Average METs were not correlated by method or with gestational age, R^2^ <0·01 (ie, participants worked at similar levels of exertion throughout pregnancy), or with PSI_MOD_, or with environmental conditions (appendix pp 5–10).

**Figure 3:**
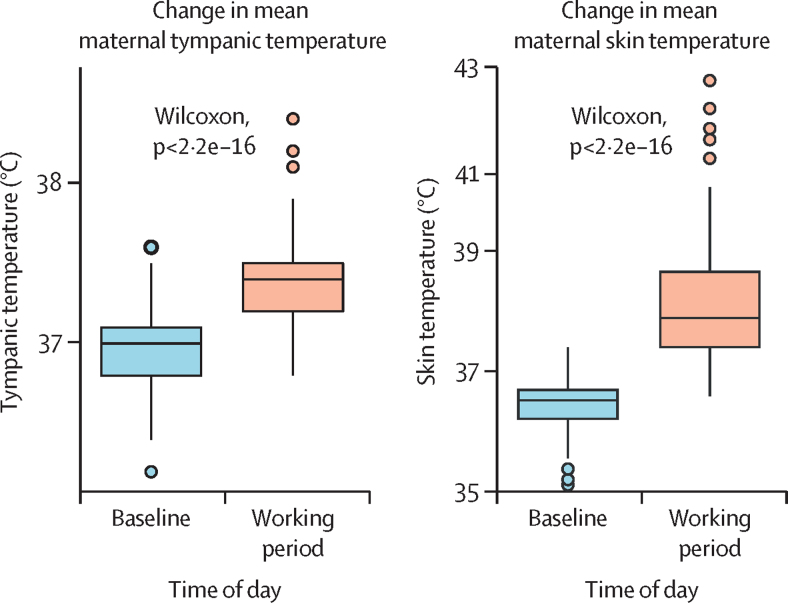
Mean change in maternal tympanic and skin temperature from baseline to the working period

There was a significant increase in both mean tympanic and mean skin temperature from baseline to the working period (p<0·0001; figure 3). Maternal heart rate also increased from baseline to the working period (p<0·0001). The mean peak PSI_MOD_ was 5·1 (SD 2·4). There was a clear linear association between heat stress and heat strain (PSI_MOD_, appendix p 11).

Symptoms of heat illness were common in this cohort, with 71 (58%) of 122 participants experiencing at least one symptom during field visits. The most commonly reported symptom was dry mouth (37%), followed by headache (21%) and muscle cramps (17%).

There was a significant increase in FHR from baseline at rest (125 [SD  7·9] bpm) to the working period (147 [SD 11·9] bpm; p<0·0001; appendix p 12). During field visits, a total of 41 (34%) of 122 episodes of fetal strain were measured. 21 participants had a FHR greater than or equal to 160 or less than 115 bpm. In participants who were eligible for UmbiFlow (n=40), resistance index changes by category occurred in 22 (55%) of 40 participants from baseline. All of these participants were monitored for return to baseline measurements after 30 min. All but one participant returned to baseline, and that participant was subsequently referred for urgent antenatal care. Despite close follow-up care, this patient went on to have a stillbirth.

**Figure 4:**
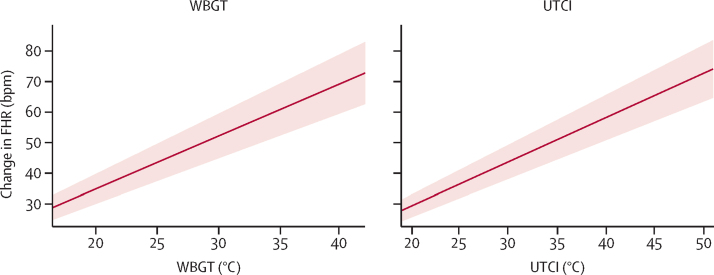
Adjusted association between change in FHR and heat stress exposure FHR=fetal heart rate. bpm=beats per min. WBGT=wet bulb globe temperature. UTCI=universal thermal climate index.

In multivariable analyses, both heat stress indices were similar (with similar Akaike information criterion) and were significantly associated with both fetal heart rate and fetal strain both in model 1 (total effect) and in model 2 (controlling for maternal heat strain; table 2). Figure 4 shows the total effect between FHR and the UTCI or WBGT. When heat stress was defined by the UTCI, total effect on fetal strain resulted in an OR of 1·17 (95% CI 1·09–1·29; p<0·0001), and an adjusted direct effect of OR of 1·12 (1·03–1·21; p=0·010) with each 1°C increase in UTCI. When heat stress was defined by WBGT, total effect of heat stress on fetal strain resulted in an OR of 1·20 (1·12–1·29; p<0·0001), with an adjusted direct effect of OR of 1·12 (1·02–1·24; p=0·018) with each 1°C increase in WBGT. Maternal heat strain was significantly associated with fetal strain when adjusted for heat stress, percentage fat mass, fitness, and gestational age (in the UTCI model, OR 1·20 [1·01–1·43; p=0·038]; in the WBGT model, OR 1·22 [1·03–1·46, p=0·025]), with each unit increase in PSI_MOD_.

**Table 2:** Multivariable repeated measure models of fetal heart rate and fetal strain

	**Model 1**		**Model 2**		**Model 3**	
	Estimate (95% CI)	p value	Estimate (95% CI)	p value	Estimate (95% CI)	p value
**Model A1—fetal heart rate and UTCI**						
UTCI	1·45 (1·26–1·64)	<0·0001	1·18 (0·87–1·49)	<0·0001	1·19 (0·88–1·49)	<0·0001
PSI	1827·2	..	0·77 (0·06–1·47)	0·033	0·82 (0·11–1·52)	0·025
AIC	..	..	1825·0	..	1824·8	..
**Model A2—fetal heart rate and WBGT**						
WBGT	1·74 (1·50–1·98)	<0·0001	1·26 (0·87–1·65)	<0·0001	1·27 (0·88–1·66)	<0·0001
PSI	1845·2	..	1·11 (0·39–1·82)	0·0024	1·17 (0·45–1·88)	0·0015
AIC	..	..	1838·3	..	1838·1	..
**Model B1—fetal strain and UTCI**						
UTCI	OR 1·17 (1·09–1·29)	<0·0001	OR 1·12 (1·03–1·21)	0·010	OR 1·13 (1·03–1·23)	0·0090
PSI	..	..	OR 1·18 (1·01–1·37)	0·042	OR 1·20 (1·01–1·43)	0·038
AIC	194·0	..	191·6	..	189·7	..
**Model B2—fetal strain and WBGT**						
WBGT	OR 1·20 (1·12–1·29)	<0·0001	OR 1·12 (1·02–1·24)	0·018	OR 1·14 (1·02–1·26)	0·016
PSI	..	..	OR 1·19 (1·02–1·40)	0·031	OR 1·22 (1·03–1·46)	0·025
AIC	195·6	..	192·7	..	190·9	..

Model 1 represents the total effect of heat stress on heat strain. Model 2 represents the direct effect of heat stress on fetal strain, controlling for maternal heat strain. Model 3 represents the direct effect of heat strain on fetal strain, controlling for heat stress, percentage fat mass, fitness, and gestational age. UTCI=universal thermal climate index (heat stress). PSI=physiological strain index (heat strain). AIC=Akaike information criterion. WBGT=wet bulb globe temperature (heat stress). OR=odds ratio.

## Discussion

To the best of our knowledge, this is the first study to explore the dynamic changes to maternal and fetal physiology in pregnant farmers under extreme environmental heat stress, or indeed pregnant manual workers of any description. We found that exposure to extreme temperatures beyond the recommended outdoor working limits were a common occurrence for pregnant subsistence farmers in our study region (West Kiang) of The Gambia. We found a strong association between maternal heat strain and heat stress exposure. The total and adjusted direct effects of maternal heat stress exposure were significantly associated with fetal strain. Maternal heat strain, controlling for heat stress exposure, was also significantly associated with fetal strain. These findings indicate that, although maternal heat strain is likely to be on the pathway from heat stress to fetal stress, there are possibly other important biological mechanisms occurring that need further exploration.

The existing literature focuses on heat thresholds during early pregnancy, as heat can act as a teratogen, and applies the established threshold of 39·5°C core temperature to the whole pregnancy. However, the mechanisms by which heat can adversely affect outcomes throughout pregnancy and core temperature thresholds in the second and third trimesters have not been established. We provide evidence to show that maternal tympanic membrane temperature increases while doing manual tasks in the heat, even in only moderate intensity activities (metabolic equivalent 3–6), but did not exceed 38·5°C. Despite this modest rise in body temperature, we found evidence of physiological strain in both mother and fetus. We suggest that an important physiological factor to consider in future work is the role of the diversion of blood from the placenta to the skin, which occurs at lower core temperatures than those that have been previously highlighted as teratogenic. This factor has been well recognised in maternal exercise and has been signposted as a potential issue in maternal thermoregulation, but has not been thoroughly investigated.^32^, ^33^

Although this study was not designed to specifically evaluate the applicability of heat stress indices in pregnancy, both UTCI and WBGT models performed similarly, which could aid the accessibility and generalisability of our data for future studies. Few studies have explored the effects of heat on fetal physiology, although the literature is more extensive on the effects of exercise.^16^ Heat stress from saunas has been shown to lead to an increase in FHR, although no studies in saunas have led to increases in FHR above 160 bpm and none have shown changes to the umbilical artery resistance index.^34^ Our finding that a total of 41 (34%) of 122 episodes of fetal strain were measured was high. However, heat exposure levels in our population were very high and these levels are similar to levels of heat strain in workers in other occupational settings.^11^ The dearth of comparable studies highlights the ongoing need to focus on this area.

Heat protection of agricultural workers is of growing concern due to the climate crisis and adaptation strategies are being explored as a matter of urgency.^35^ However, subsistence farmers are mostly missing from these strategies. Our findings of self-reported symptoms of heat illness in 58% of participants is similar to the levels found by Frimpong and colleagues^36^ in their study of smallholder farmers in Ghana, and Sadiq and colleagues’^37^ study of maize farmers in Nigeria, but higher than that found in Flouris and colleagues’^11^ systematic review. A study of pregnant farm workers in Florida (USA) reported that there were high rates of heat illness symptoms in these workers and a lack of agency to alter workplace conditions.^38^ For subsistence farmers, the pressures are often different, but the inability to avoid the heat is a shared problem, as detailed in our qualitative analysis of heat perception during pregnancy in this population.^39^

There are several limitations to our study. First, as discussed in the Results section, the COVID-19 pandemic had several adverse impacts on our study and reduced our sample size. Second, there were issues with gold standard measurements. In a field-based study in rural sub-Saharan Africa, a rectal thermometer was not acceptable and core telemetry pills do not yet have a safety track record in pregnancy and therefore could not be used. We used tympanic temperature while recognising its flaws, but aimed to minimise these by taking duplicate measurements from each participants’ ear at all timepoints. Triaxial accelerometery and heart rate monitoring to estimate energy expenditure in pregnancy is less accurate than in other populations. The PSI is also not validated in pregnancy, where concerns for both mother and fetus must be considered. To aid generalisability and applicability, future studies should aim to establish the accuracy of the PSI during pregnancy. Additionally, this population was exposed to high levels of heat stress throughout the study, which is likely to have resulted in acclimatisation and also prevented comparisons to groups with less heat exposure. Lastly, our population had poorer birth outcomes than the global average, which might have affected the generalisability of our findings.

Globally, there is a large population at risk to extreme heat, with future predictions being exceedingly concerning. Extreme heat exposure, which poses a risk to health from even moderate physical labour in non-pregnant adults during the hottest month of the year, is predicted to affect 350 million people by the year 2100 if the Paris Agreement of limiting temperature rise to 1·5°C is met, which is increasingly unlikely. Should temperature increases reach 2·5°C above pre-industrial levels, this scenario will mean that approximately 1 billion people will be exposed to dangerous levels of heat stress.^40^ There remain large evidence gaps in relation to the pathophysiology of heat in pregnancy, the identification of those most at risk, and the development of suitable and effective interventions to reduce adverse birth outcomes. Further work in a variety of settings and populations that explore the changes in placental blood flow following heat stress, as well as the association with pregnancy outcomes and heat stress, is urgently needed. In addition, the co-development of trials in pregnant subsistence farmers in sub-Saharan Africa is an urgent imperative.

## Data sharing

Anonymised data will be made available by the corresponding author upon reasonable request.

## Declaration of interests

We declare no competing interests.

## References

[1] World Meteorological Organization (2021). The state of the global climate 2020. https://public.wmo.int/en/our-mandate/climate/wmo-statement-state-of-global-climate.

[2] Watts N, Amann M, Arnell N (2021). The 2020 report of the *Lancet* Countdown on health and climate change: responding to converging crises. Lancet.

[3] Bekkar B, Pacheco S, Basu R, DeNicola N (2020). Association of air pollution and heat exposure with preterm birth, low birth weight, and stillbirth in the US: a systematic review. JAMA Netw Open.

[4] Chersich MF, Pham MD, Areal A (2020). Associations between high temperatures in pregnancy and risk of preterm birth, low birth weight, and stillbirths: systematic review and meta-analysis. BMJ.

[5] Samuels L, Nakstad B, Roos N (2022). Physiological mechanisms of the impact of heat during pregnancy and the clinical implications: review of the evidence from an expert group meeting. Int J Biometeorol.

[6] King AD, Harrington LJ (2018). The inequality of climate change from 1.5 to 2°C of global warming. Geophys Res Lett.

[7] Ebi KL, Capon A, Berry P (2021). Hot weather and heat extremes: health risks. Lancet.

[8] Sylla MB, Nikiema PM, Gibba PKI, Kluste NAB, Yjh J (2016). Adaptation to climate change and variability in rural west Africa.

[9] Grace K, Davenport F, Hanson H, Funk C, Shraddhanand S (2015). Linking climate change and health outcomes: examining the relationship between temperature, precipitation and birth weight in Africa. Glob Environ Change.

[10] The Gambia Bureau of Statistics (2018). The Gambia Labour Force Survey. https://www.gbosdata.org/downloads/gambia-labour-force-survey-29.

[11] Flouris AD, Dinas PC, Ioannou LG (2018). Workers’ health and productivity under occupational heat strain: a systematic review and meta-analysis. Lancet Planet Health.

[12] Yates DT, Petersen JL, Schmidt TB (2018). ASAS-SSR triennnial reproduction symposium: looking back and moving forward—how reproductive physiology has evolved: fetal origins of impaired muscle growth and metabolic dysfunction: lessons from the heat-stressed pregnant ewe. J Anim Sci.

[13] Ziegert M, Witkin SS, Sziller I, Alexander H, Brylla E, Härtig W (1999). Heat shock proteins and heat shock protein-antibody complexes in placental tissues. Infect Dis Obstet Gynecol.

[14] Edwards MJ (1986). Hyperthermia as a teratogen: a review of experimental studies and their clinical significance. Teratog Carcinog Mutagen.

[15] Smallcombe JW, Puhenthirar A, Casasola W (2021). Thermoregulation during pregnancy: a controlled trial investigating the risk of maternal hyperthermia during exercise in the heat. Sports Med.

[16] Ravanelli N, Casasola W, English T, Edwards KM, Jay O (2019). Heat stress and fetal risk. Environmental limits for exercise and passive heat stress during pregnancy: a systematic review with best evidence synthesis. Br J Sports Med.

[17] Hennig BJ, Unger SA, Dondeh BL (2017). Cohort profile: the Kiang West Longitudinal Population Study (KWLPS)—a platform for integrated research and health care provision in rural Gambia. Int J Epidemiol.

[18] Bonell A, Hirst J, Vicedo-Cabrera AM, Haines A, Prentice AM, Maxwell NS (2020). A protocol for an observational cohort study of heat strain and its effect on fetal wellbeing in pregnant farmers in The Gambia. Wellcome Open Res.

[19] Morabito ACM (2016). rBiometeo: biometeorological functions in R. https://rdrr.io/github/alfcrisci/rBiometeo/.

[20] Havenith G, Fiala D (2015). Thermal indices and thermophysiological modeling for heat stress. Compr Physiol.

[21] Brinnel H, Cabanac M (1989). Tympanic temperature is a core temperature in humans. J Therm Biol.

[22] Yeoh WK, Lee JKW, Lim HY, Gan CW, Liang W, Tan KK (2017). Re-visiting the tympanic membrane vicinity as core body temperature measurement site. PLoS One.

[23] Moran DS, Shitzer A, Pandolf KB (1998). A physiological strain index to evaluate heat stress. Am J Physiol.

[24] Eggenberger P, MacRae BA, Kemp S, Bürgisser M, Rossi RM, Annaheim S (1780). Prediction of core body temperature based on skin temperature, heat flux, and heart rate under different exercise and clothing conditions in the heat in young adult males. Front Physiol.

[25] van Hees VT, Renström F, Wright A (2011). Estimation of daily energy expenditure in pregnant and non-pregnant women using a wrist-worn tri-axial accelerometer. PLoS One.

[26] Brage S, Ekelund U, Brage N (2007). Hierarchy of individual calibration levels for heart rate and accelerometry to measure physical activity. J Appl Physiol (1985).

[27] Lawrence M, Singh J, Lawrence F, Whitehead RG (1985). The energy cost of common daily activities in African women: increased expenditure in pregnancy?. Am J Clin Nutr.

[28] Centers for Disease Control and Prevention (2017). Heat-related illness. http://www.cdc.gov/disasters/extremeheat/warning.html.

[29] Nguyen NC, Evenson KR, Savitz DA, Chu H, Thorp JM, Daniels JL (2013). Physical activity and maternal-fetal circulation measured by Doppler ultrasound. J Perinatol.

[30] Hlongwane T, Cronje T, Nkosi B, Pattinson RC (2021). The prevalence of abnormal Doppler's of the umbilical artery in a low-risk pregnant population in South Africa. EClinicalMedicine.

[31] Gasparrini A (2011). Distributed lag linear and non-linear models in R: the package dlnm. J Stat Softw.

[32] Ertan AK, Schanz S, Tanriverdi HA, Meyberg R, Schmidt W (2004). Doppler examinations of fetal and uteroplacental blood flow in AGA and IUGR fetuses before and after maternal physical exercise with the bicycle ergometer. J Perinat Med.

[33] Ziskin MC, Morrissey J (2011). Thermal thresholds for teratogenicity, reproduction, and development. Int J Hyperthermia.

[34] Vähä-Eskeli K, Pirhonen J, Seppänen A, Erkkola R (1991). Doppler flow measurement of uterine and umbilical arteries in heat stress during late pregnancy. Am J Perinatol.

[35] Ioannou LG, Mantzios K, Tsoutsoubi L (2021). Occupational heat stress: multi-country observations and interventions. Int J Environ Res Public Health.

[36] Frimpong K, Odonkor ST, Kuranchie FA, Nunfam VF (2020). Evaluation of heat stress impacts and adaptations: perspectives from smallholder rural farmers in Bawku East of northern Ghana. Heliyon.

[37] Sadiq LS, Hashim Z, Osman M (2019). The impact of heat on health and productivity among maize farmers in a tropical climate area. J Environ Public Health.

[38] Flocks J, Vi Thien Mac V, Runkle J, Tovar-Aguilar JA, Economos J, McCauley LA (2013). Female farmworkers’ perceptions of heat-related illness and pregnancy health. J Agromed.

[39] Spencer S, Samateh T, Wabnitz K, Mayhew S, Allen H, Bonell A (2022). The challenges of working in the heat whilst pregnant: insights from Gambian women farmers in the face of climate change. Front Public Health.

[40] Andrews O, Le Quéré C, Kjellstrom T, Lemke B, Haines A (2018). Implications for workability and survivability in populations exposed to extreme heat under climate change: a modelling study. Lancet Planet Health.

