# Correction to *Lancet Planet Health* 2022; 6: e968–76

**DOI:** 10.1016/S2542-5196(23)00167-5

**Published:** 2023-07-15

*Bonell A, Sonko B, Badjie J, et al. Environmental heat stress on maternal physiology and fetal blood flow in pregnant subsistence farmers in The Gambia, west Africa: an observational cohort study.* Lancet Planet Health *2022; **6:** e968–76*—In this Article, the wet bulb temperature readings were found to be inaccurate owing to a malfunctioning device. We have updated analyses related to the wet bulb globe temperature measurements based on an alternative sensor and made corrections to the summary findings, the main methods and results sections, figures 2 and 4, tables, and the discussion. No total effect or OR was reduced by more than 0·27. The appendix has also been corrected. These corrections have been made to the online version as of July 17, 2023.

