# Supplementary material for: Environmental heat stress on maternal physiology and fetal blood flow in pregnant subsistence farmers in The Gambia, west Africa: an observational cohort study
Source: Lancet Planet Health. 2022 Dec 7;6(12):e968–76. doi: 10.1016/S2542-5196(22)00242-X (PMC9756110; doi:10.1016/S2542-5196(22)00242-X)
Supplement: Supplementary appendix [file mmc1.pdf]

THE LANCET
Planetary Health

Supplementary appendix

This appendix formed part of the original submission and has been peer reviewed. We post it as supplied by the authors.

Supplement to: Bonell A, Sonko B, Badjie J, et al. Environmental heat stress on maternal physiology and fetal blood flow in pregnant subsistence farmers in The Gambia, west Africa: an observational cohort study. *Lancet Planet Health* 2022; **6:** e968–76.

SUPPLEMENT

A cohort study assessing the effect of environmental heat stress on maternal physiology and fetal blood flow in pregnant subsistence farmers in The Gambia, West Africa

Authors: Ana Bonell, Bakary Sonko, Jainaba Badjie, Tida Samateh, Tida Saidy, Fatou Sosseh, Yahya Sallah, Kebba Bajo, Kris A Murray, Jane Hirst, Ana Vicedo-Cabrera, Andrew M Prentice, Neil S Maxwell, Andy Haines

Table of contents

Table 1: univariate analyses of PSI_{MOD}, fetal heart rate and fetal stress……………………………….. 2
Figure 1: Flow-chart of study recruitment………………………………………………………… 3
Figure 2: Average Temperature recorded per field visit.. 4
Figure 3: Daily temperature recorded from the closest weather station (Kerewan) 2019…..………….. 4
Figure 4: Metabolic equivalent (MET) comparison: method A against method B…………………….. 5
Figure 5a: MET (method A) against WBGT exposure……………………………………………. 5
Figure 5b: MET (method A) against UTCI exposure……………………………………………... 6
Figure 5c: MET (method A) against modified physiological strain index (PSI_{MOD})…………………… 6
Figure 5d: MET (method B) against gestational age at field visit in weeks……………………………. 7
Figure 6a: MET (method B) against WBGT exposure……………………………………………. 7
Figure 6b: MET (method B) against UTCI exposure……………………………………………... 8
Figure 6c: MET (method B) against modified physiological strain index (PSI_{MOD})…………………… 8
Figure 6d: MET (method B) against gestational age at field visit in weeks……………………………. 9
Figure 7: Correlation between environmental and physiological variables……………………………. 10
Figure 8: Association between heat stress and heat strain………..……………………………………. 11
Figure 9: Fetal heart rate (BPM) from rest to during a working shift…………………………………... 12
Figure 10: Directed Acyclic Graph (DAG) of heat stress and fetal strain……………………………… 13
Figure 11: Directed Acyclic Graph (DAG) of maternal heat strain and fetal strain……………………. 14
R code……………………………………………………………………………………………... 14

Table 1: univariate analyses of PSI_{MOD}, fetal heart rate and fetal stress

	Peak PSI_{MOD}		Fetal heart rate (bpm)		Fetal Stress (Y/N)	
	Estimate (CI)	p-value	Estimate (CI)	p-value	Odds Ratio (CI)	p-value
UTCI	0·35 (0·31;0·38)	<0·001	1·45 (1·27;1·64)	<0·001	1·17 (1·09;1·26)	<0·001
WBGT	0·43 (0·39;0·47)	<0·001	1·74 (1·50;1·98)	<0·001	1·20 (1·12;1·29)	<0·001
Air temp	0·25 (0·21;0·29)	<0·001	1·11 (0·92;1·31)	<0·001	1·11 (1·05;1·17)	<0·001
Relative humidity	-0·04 (-0·02;-0·06)	<0·001	-0·18 (-0·28;-0·08)	<0·001	0·97 (0·96;0·99)	0·01
Duration in field	0·003 (-0·003;0·01)	0·32	0·02 (-0·01;0·05)	0·18	1·00(1·00;1·01)	0·41
MET	-0·21 (-0·70;0·28)	0·41	0·63 (-1·71;2·97)	0·60	1·08 (0·72;1·64)	0·71
Tymp temp	5·48 (4·53;6·43)	<0·001	20·93 (15·99;25·86)	<0·001	7·71 (2·63;22·59)	<0·001
Skin temp	-	-	6·44 (5·24;7·63)	<0·001	1·85 (1·42;2·40)	<0·001
Heart rate	-	-	0·34 (0·29;0·38)	<0·001	1·05 (1·03;1·07)	<0·001
PSI_{MOD}	-	-	2·93 (2·46;3·40)	<0·001	1·36 (1·22;1·53)	<0·001
Osmolality	0·004 (0·002;0·006)	<0·001	0·02 (0·00;0·02)	<0·001	1·00 (1·00;1·00)	0·20
Haematocrit	-0·04 (-0·12;0·04)	0·35	-0·20 (-0·58;0·19)	0·32	0·96 (0·89;1·04)	0·37
TBW	0·04 (-0·05;0·13)	0·39	0·35 (-0·07;0·77)	0·10	1·02 (0·95;1·10)	0·58
Weight	-0·02 (-0·05;0·01)	0·24	0·03 (-0·13;0·20)	0·68	1·00 (0·95;1·10)	0·80
Maternal age	0·00 (-0·05;0·05)	0·98	0·03 (-0·22;0·28)	0·80	1·00 (0·96; 1·05)	0·75
CVS reserve	0·00 (-0·005;0·01)	0·83	6·70 (-0·02;0·03)	0·60	1·00 (1·00;1·01)	0·79
Hb	-0·11 (-0·36;0·14)	0·41	-0·46 (-1·66;0·74)	0·45	0·98 (0·93;1·03)	0·43
Fat mass	-0·06 (-0·11;-0·01)	0·02	-0·10 (-0·35;0·15)	0·43	0·98 (0·93;1·03)	0·35
GA at field visit	-0·01 (-0·06;0·05)	0·81	-0·01 (-0·29;0·26)	0·93	1·06 (1·00;1·11)	0·05
Comp in pregnancy	0·20 (-0·59;0·99)	0·62	0·74 (-3·05;4·53)	0·70	0·86 (0·45;1·71)	0·68
Adverse pregnancy outcome	0·07 (-0·73;0·86)	0·87	-1·16 (-4·96;2·64)	0·55	1·08 (0·55;2·12)	0·82

CVS reserve = cardiopulmonary reserve (measured by standard 6-minute walk test); Hb = Haemoglobin concentration; Fat mass = % fat mass as measured on bioimpedance; GA = gestational age in weeks; Comp = any complication in pregnancy except anaemia; Adverse pregnancy outcome = LBW, preterm birth, SGA, stillbirth, peripartum death.

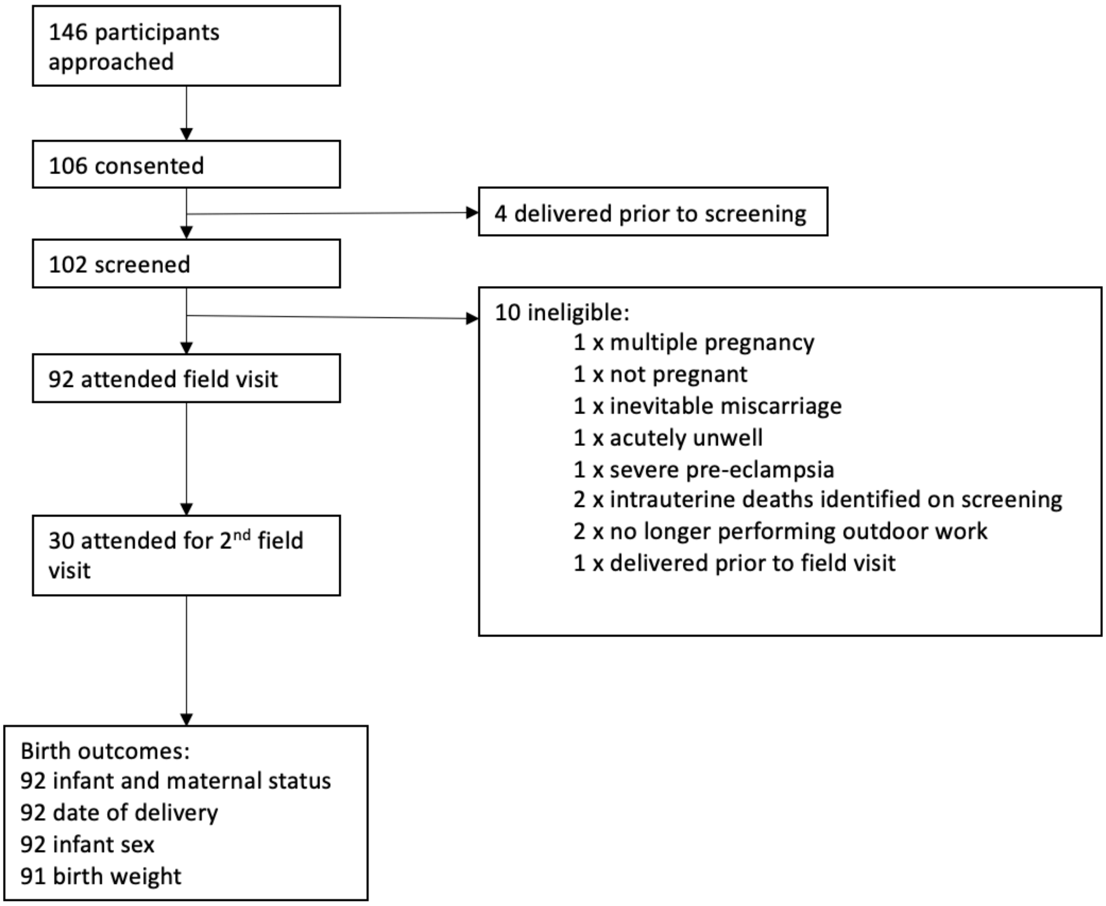

Figure 1: Flow-chart of study recruitment

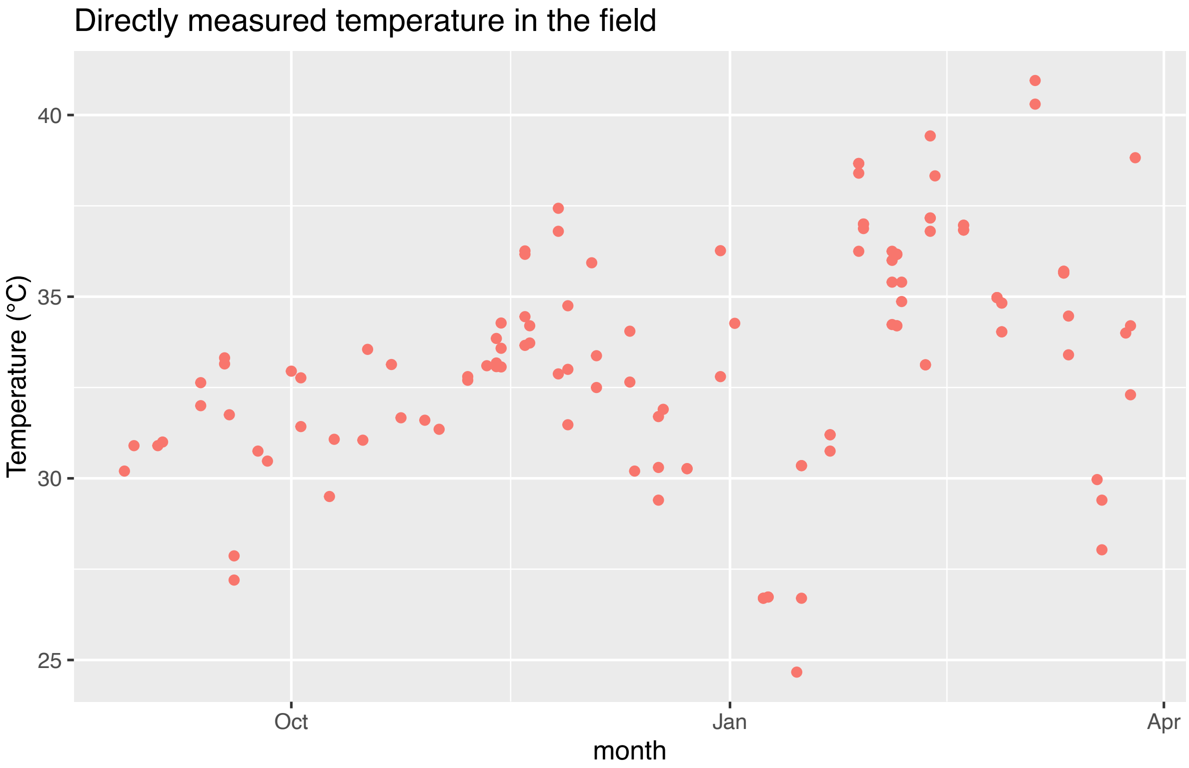

Figure 2: Directly measured temperature from each field visit (August 2019 – March 2020)

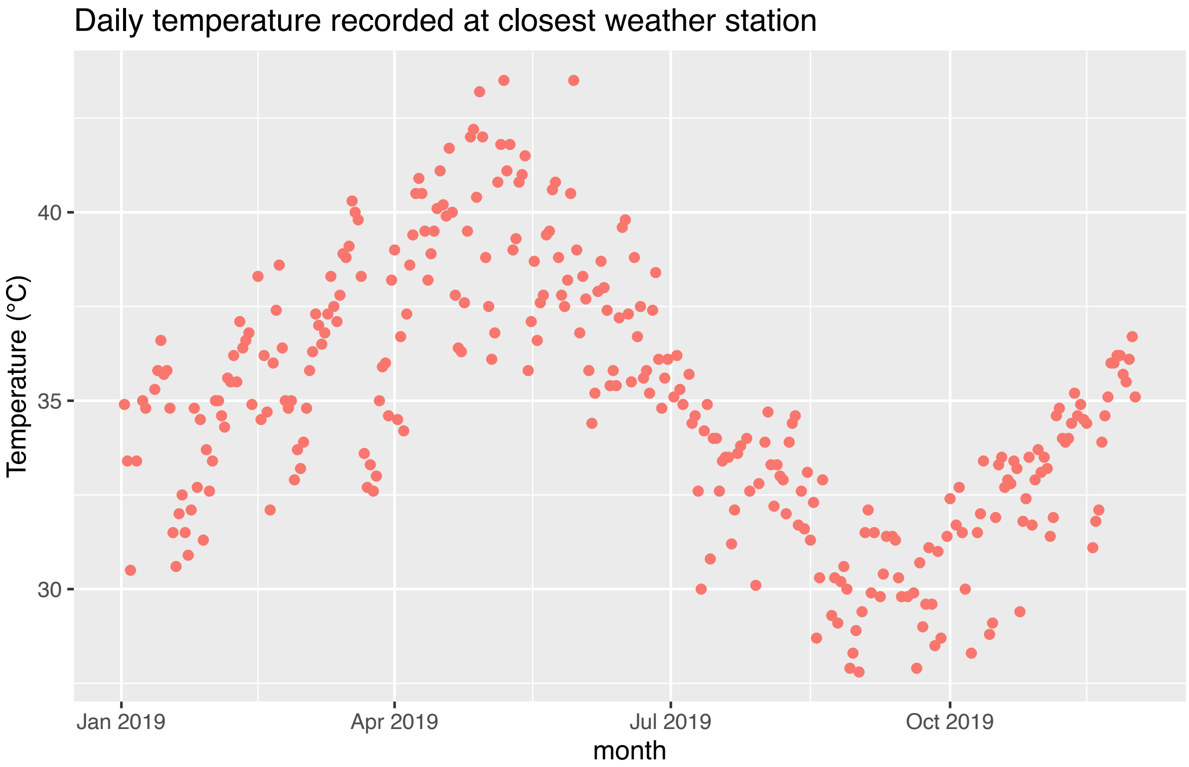

Figure 3: Daily temperature recorded at the closest weather station (Kerewan station)

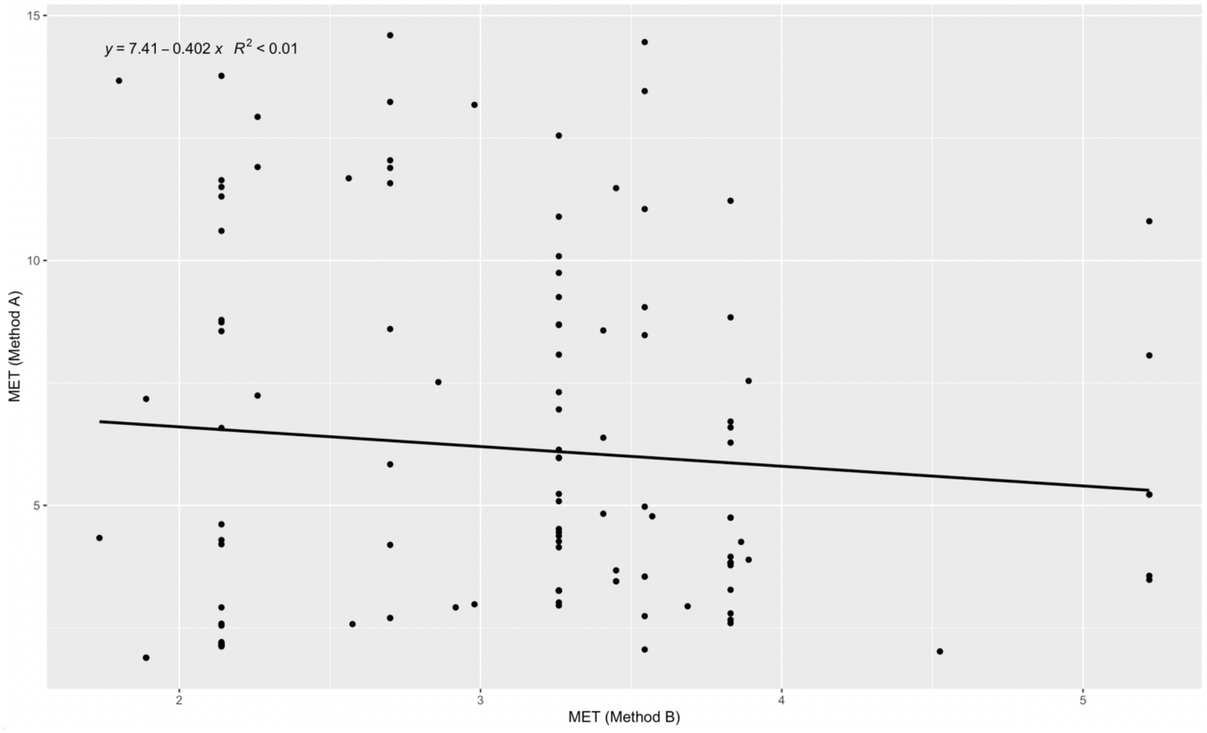

Figure 4: Metabolic equivalent (MET) comparison: method A against method B

This highlights the difficulty in estimating energy expenditure in pregnancy. Method A (calculated from continuous heart rate recorded by wearable device) and Method B (calculated from observed action in the field linked to historical measurements) are poorly correlated. Additional work on developing gestational age specific algorithms to estimate energy expenditure from wearable devices would be useful.

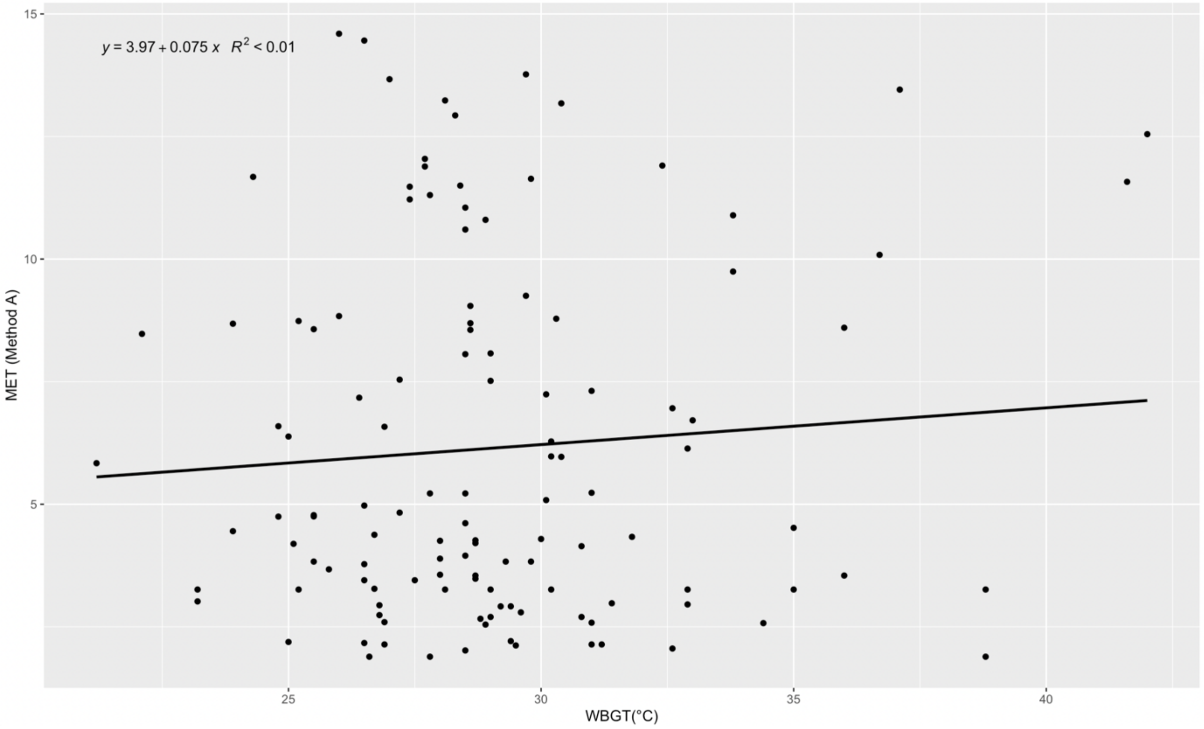

Figure 5a: MET (method A) against WBGT exposure. MET = Metabolic equivalent; WBGT = Wet Bulb Globe Temperature

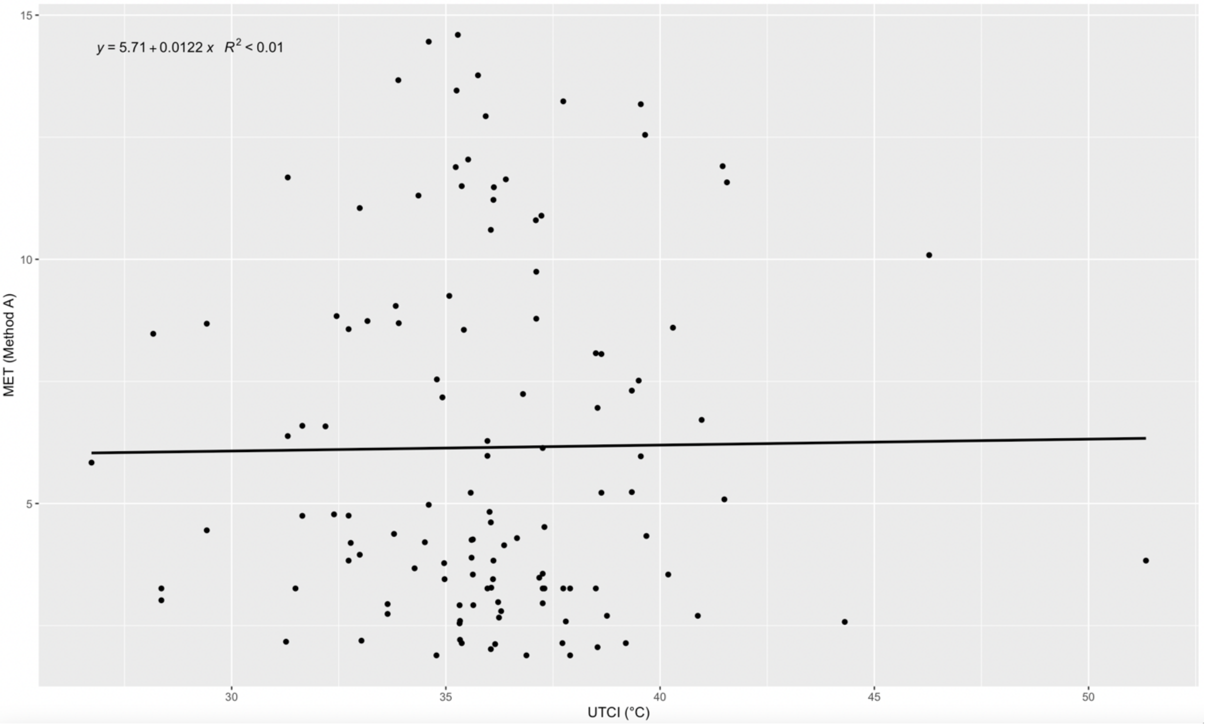

Figure 5b: MET (method A) against UTCI exposure. MET = Metabolic equivalent; UTCI = Universal Thermal Climate Index

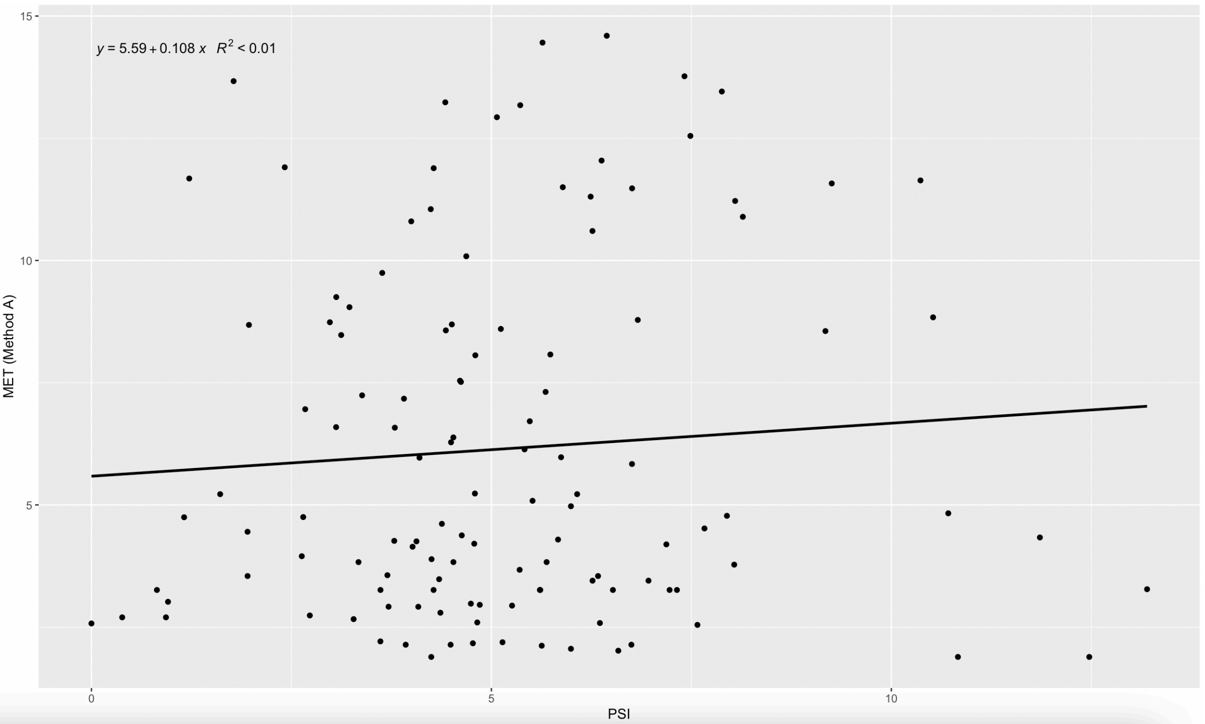

Figure 5c: MET (method A) against maternal heat strain by PSI_{MOD}. MET = Metabolic equivalent. PSI_{MOD} = modified physiological strain index

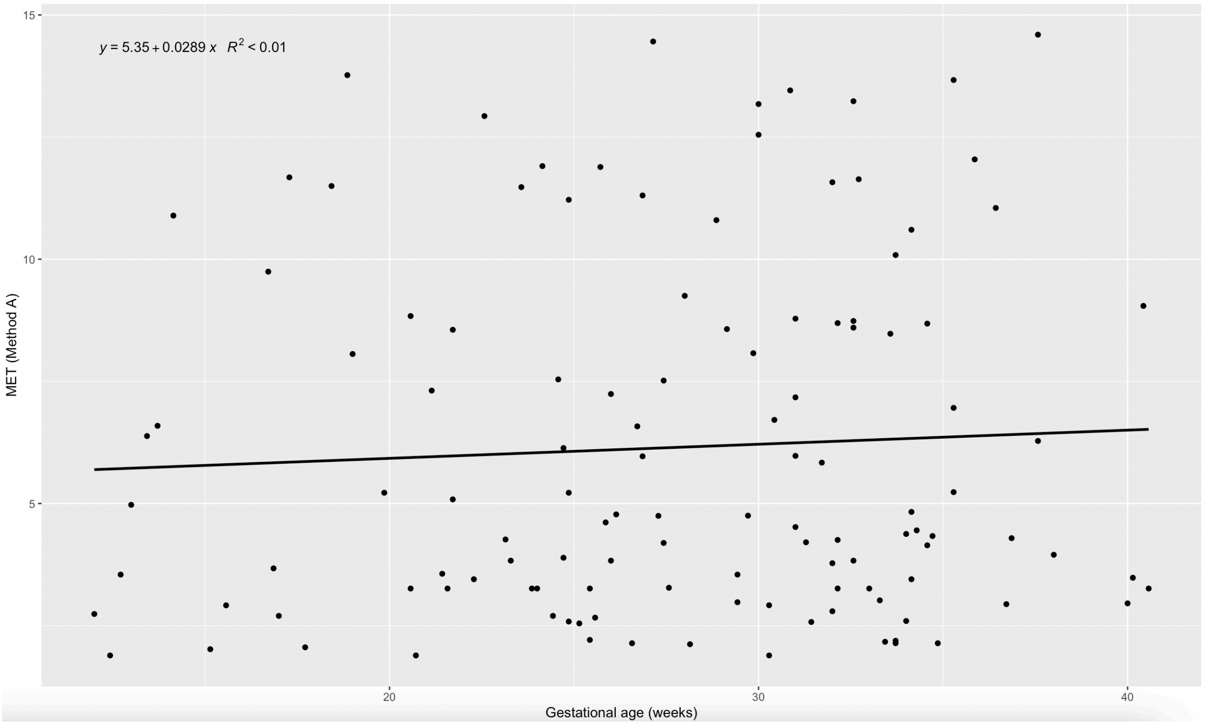

Figure 5d: MET (method A) against gestational age at field visit in weeks. MET = Metabolic equivalent

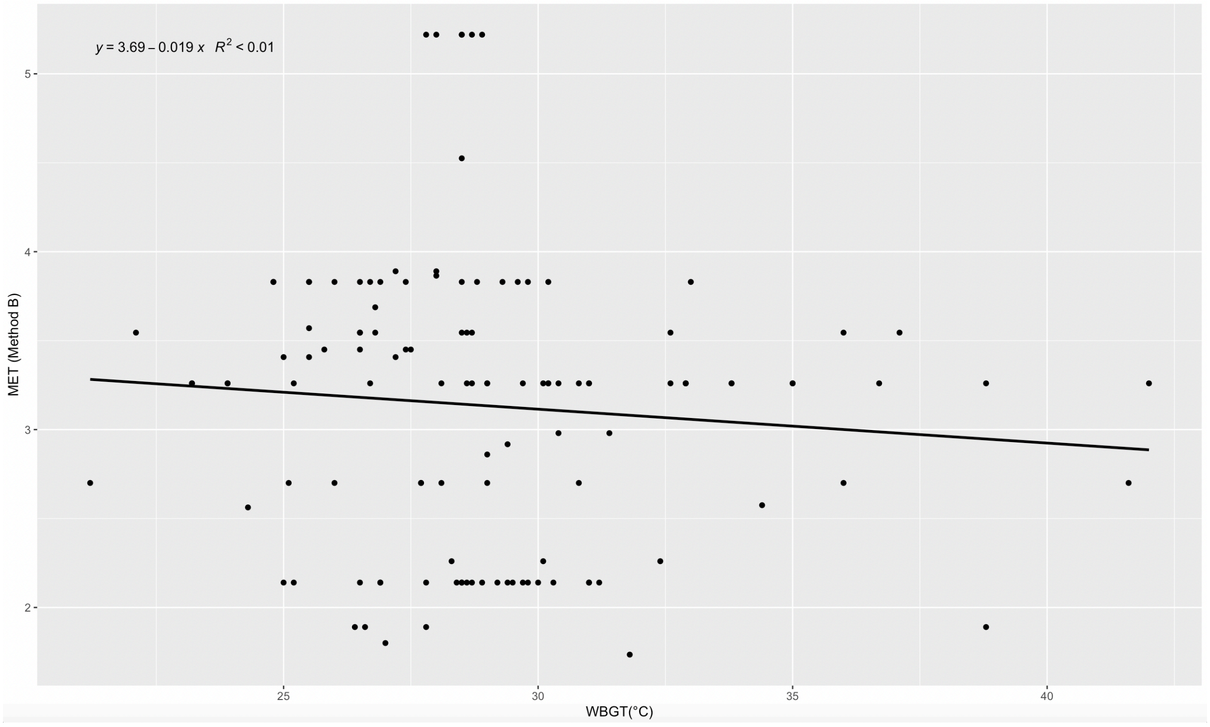

Figure 6a: MET (method B) against WBGT exposure. MET = Metabolic equivalent; WBGT = Wet Bulb Globe Temperature

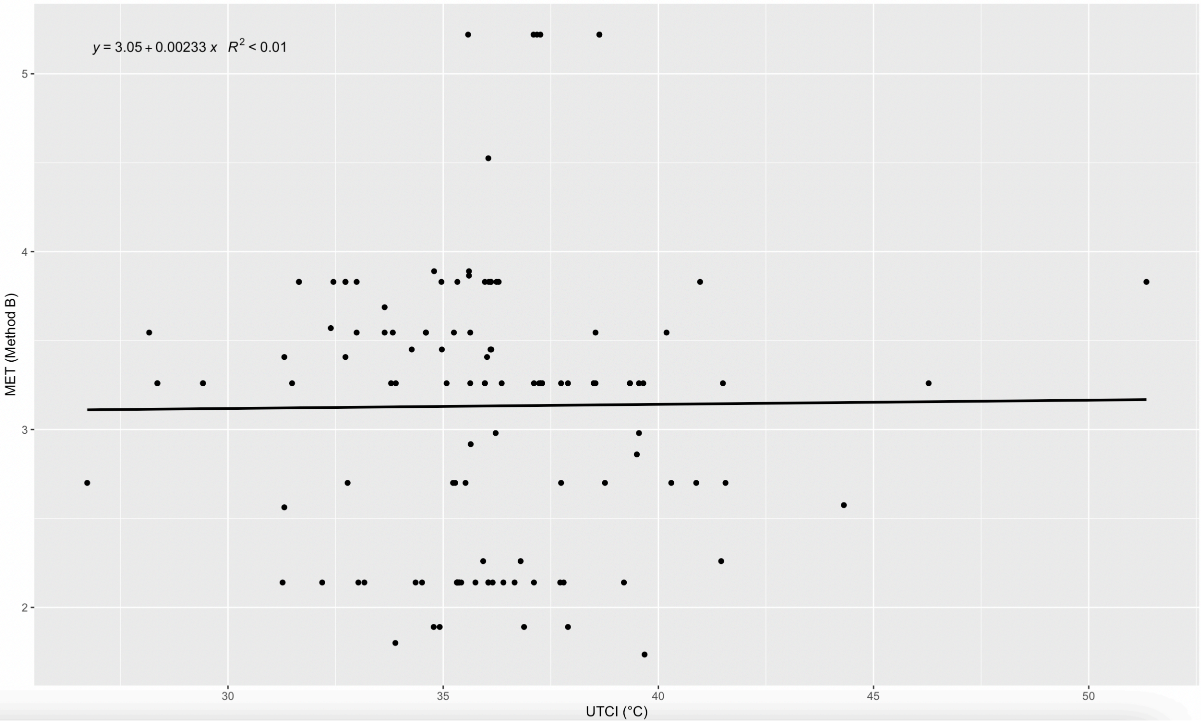

Figure 6b: MET (method B) against UTCI exposure. MET = Metabolic equivalent. UTCI = Universal Thermal Climate Index

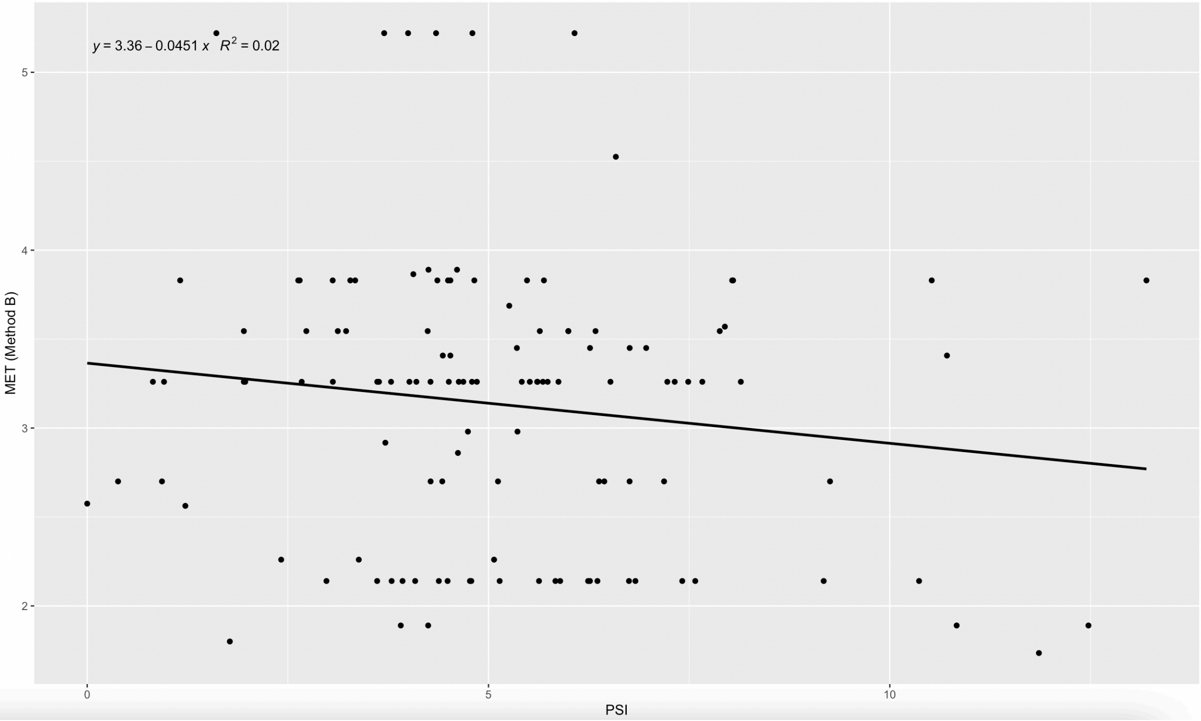

Figure 6c: MET (method B) against maternal heat strain by PSI_{MOD}. MET = Metabolic equivalent; PSI_{MOD} = modified physiological strain index

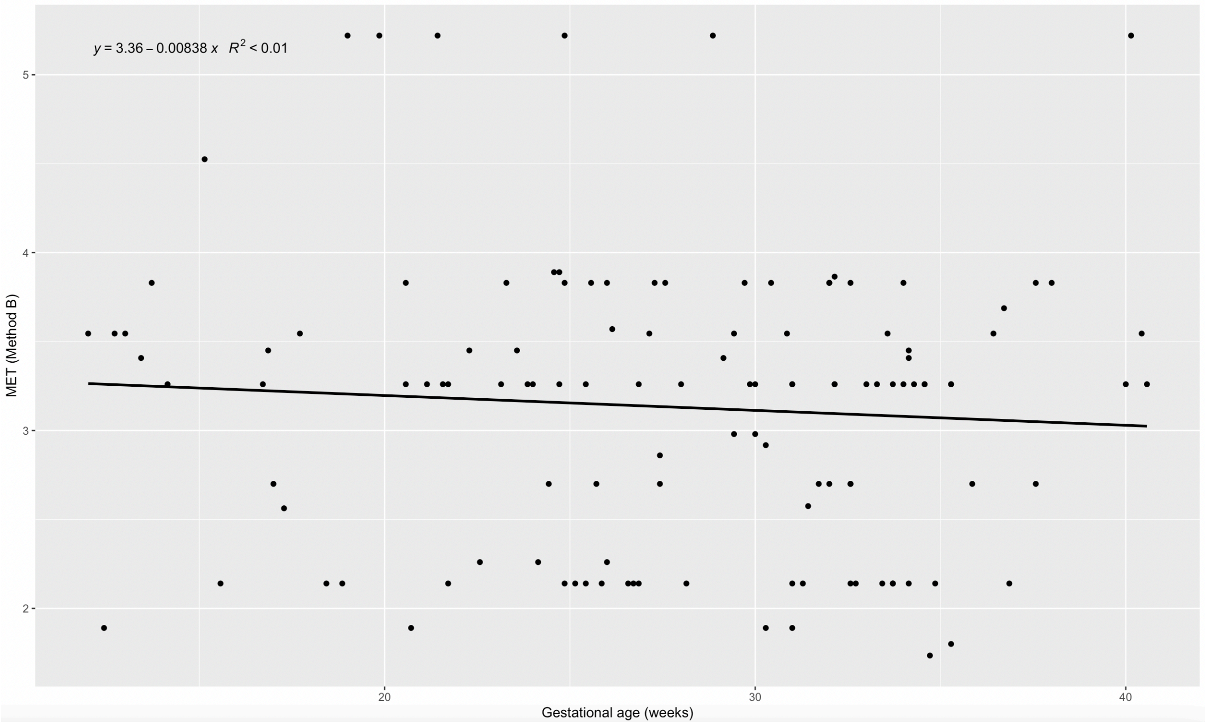

Figure 6d: MET (method B) against gestational age at field visit in weeks. MET = Metabolic equivalent

Figures 5 and 6 demonstrate the poor correlation of both our estimations of energy expenditure with environmental conditions, maternal heat strain or any changes during pregnancy. Again this highlights the need for pregnancy specific algorithms to improve accuracy of wearable technology to aid with detailed physiological understanding.

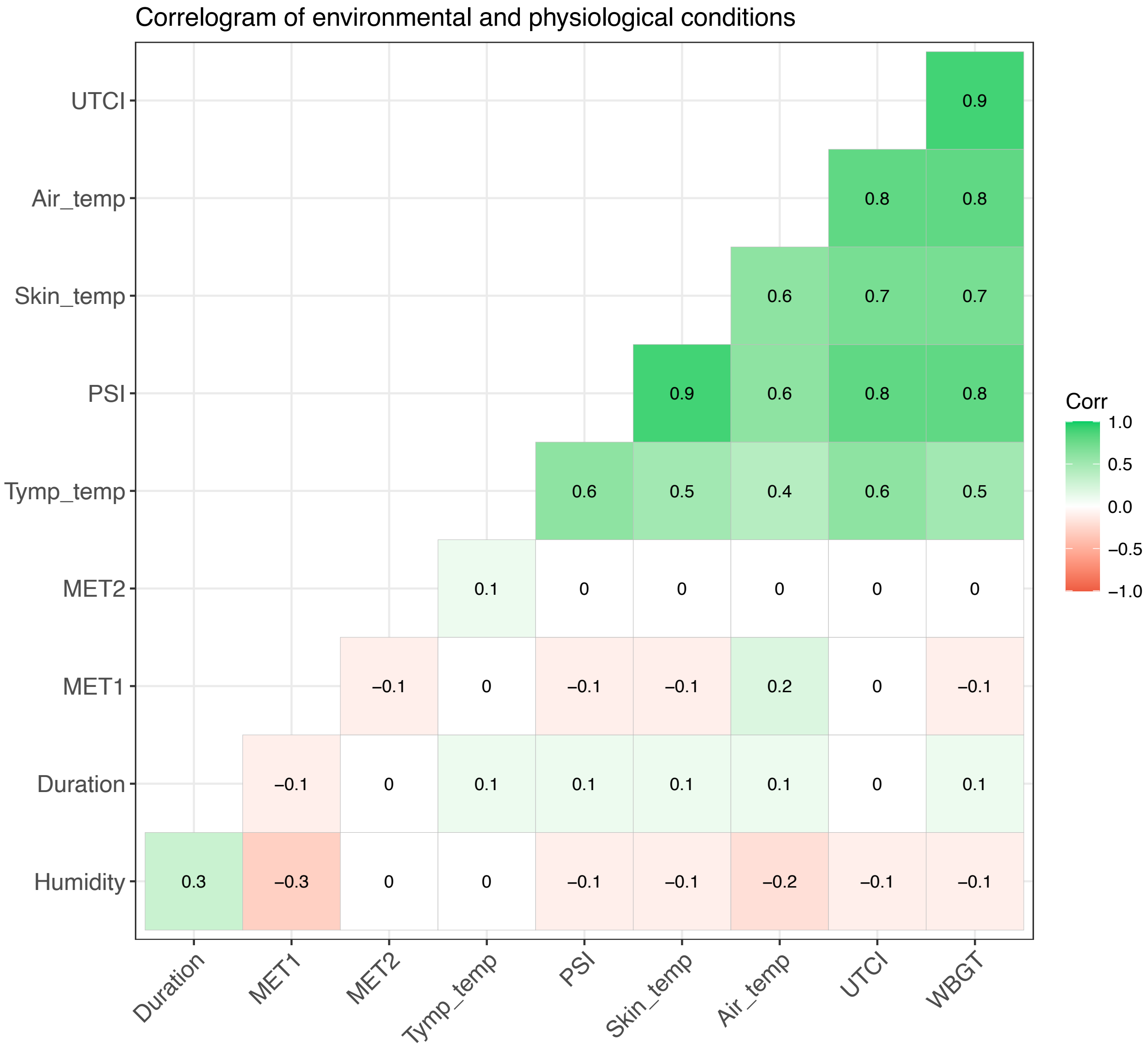

Figure 7: Pearson's correlation coefficient for environmental and physiological variables. UTCI = Universal Thermal Climate Index; WBGT = Wet Bulb Globe Temperature; air_temp = Air temperature (°C); Skin_temp = chest skin temperature (°C); PSI = modified physiological strain index; Tymp_temp = tympanic temperature (°C); MET2 = Metabolic equivalent using observation and historic measurements; MET1 = Metabolic equivalent calculated from the wearable device; Duration = time spent working outdoors; Humidity = relative humidity (%)

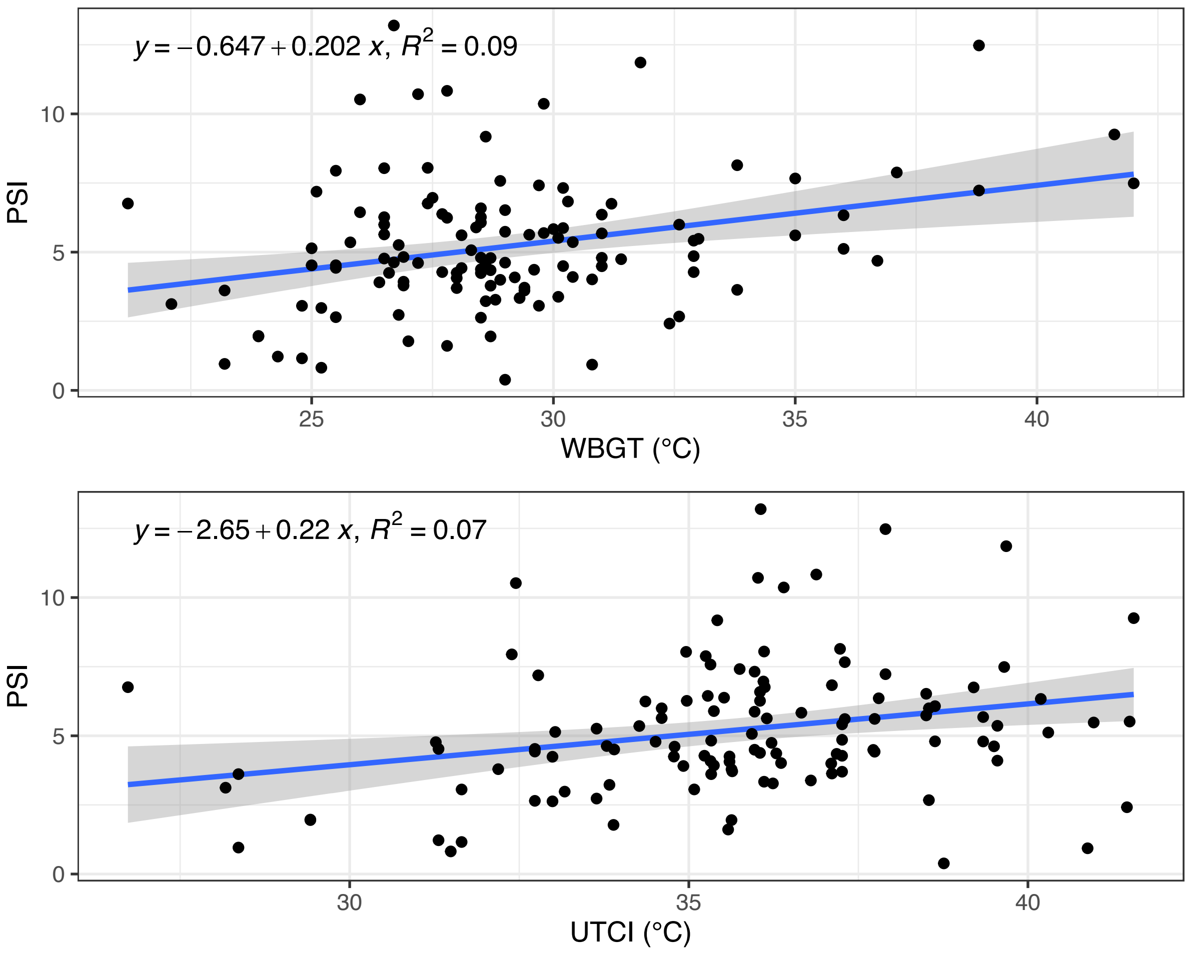

Figure 8: Association between heat stress (by WBGT and UTCI) and heat strain (by PSI_{MOD}). WBGT = Wet Bulb Globe Temperature; UTCI = Universal Thermal Climate Index; PSI_{MOD} = modified physiological strain index

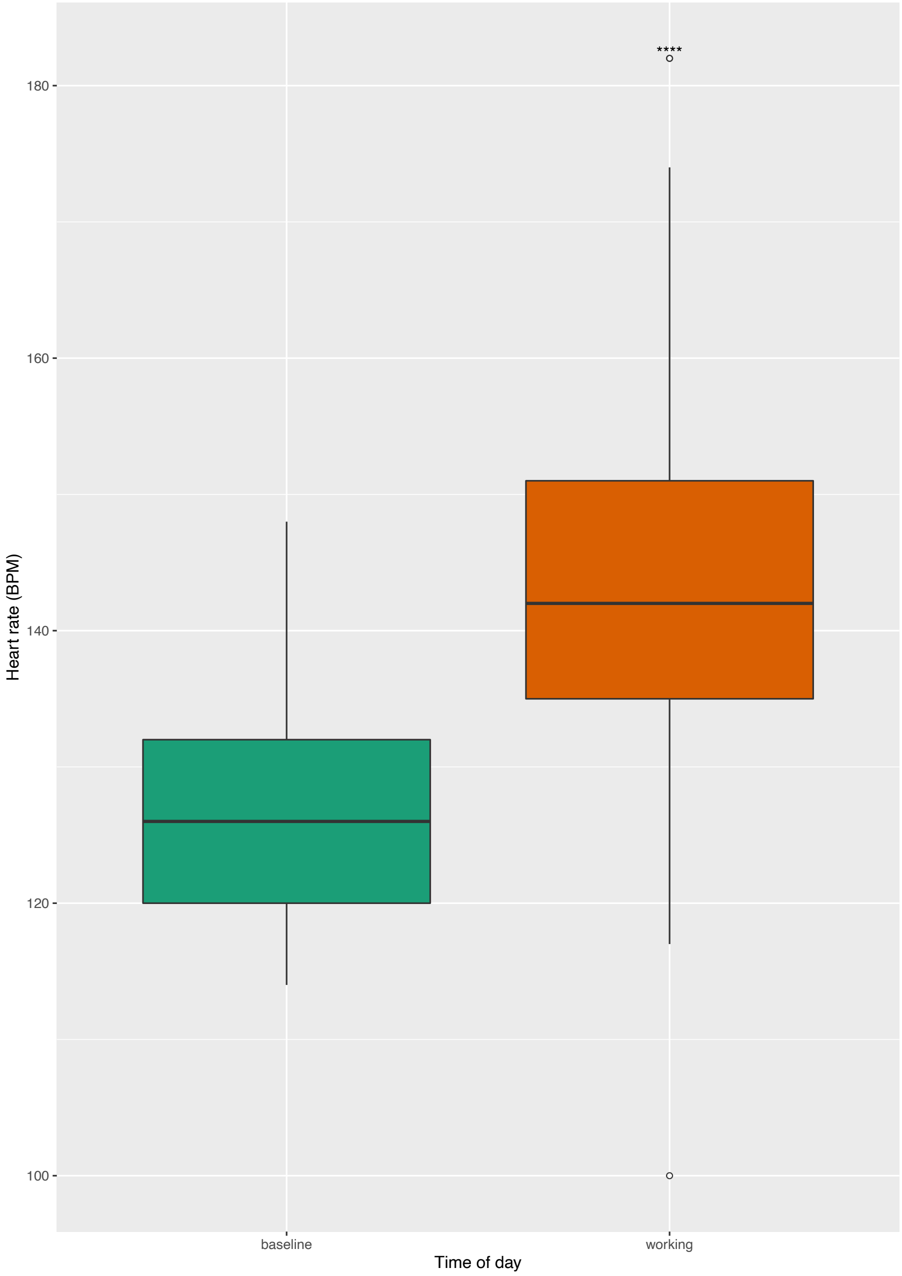

Figure 9: Change in fetal heart rate (BPM = beats per minute) from baseline to during the working shift

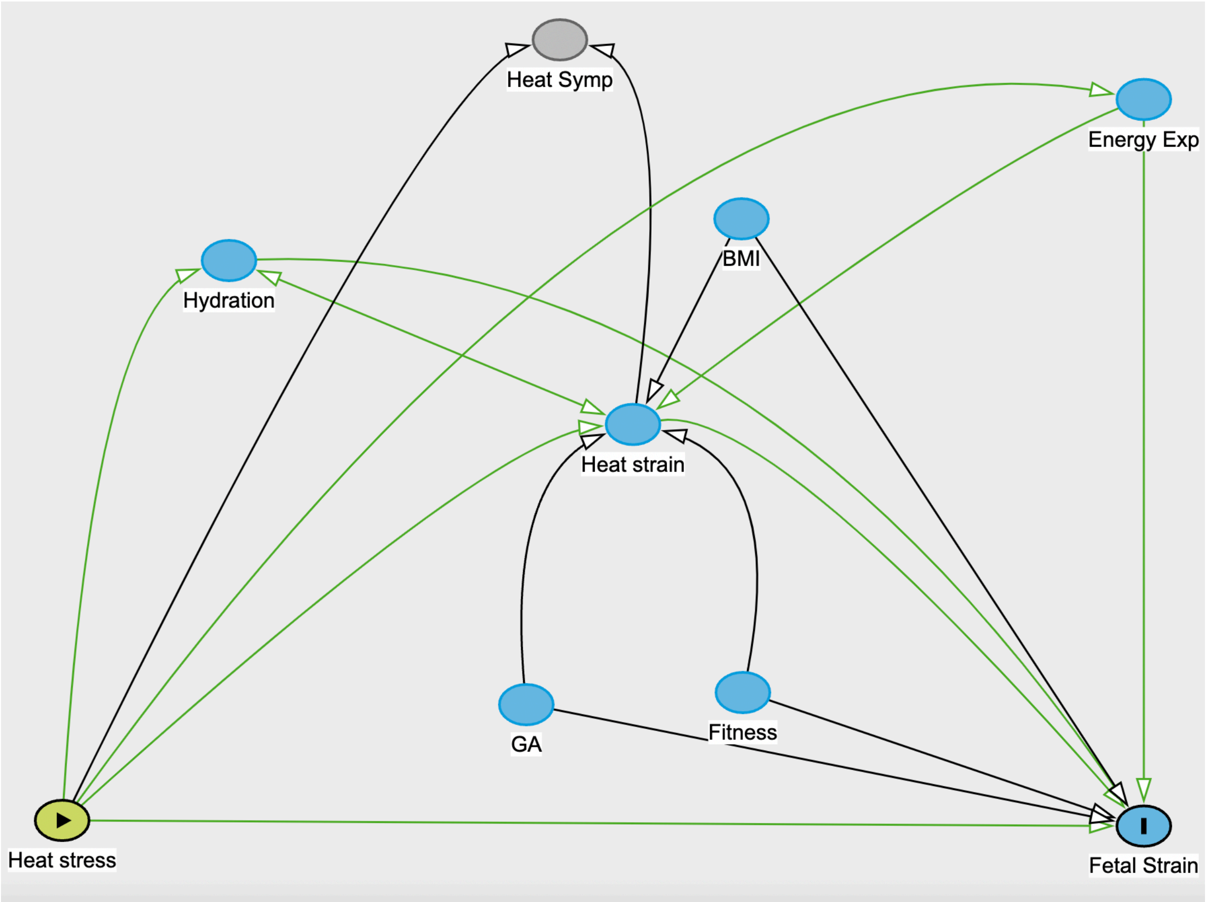

Figure 10: Directed Acyclic Graph (DAG) of heat stress and fetal strain

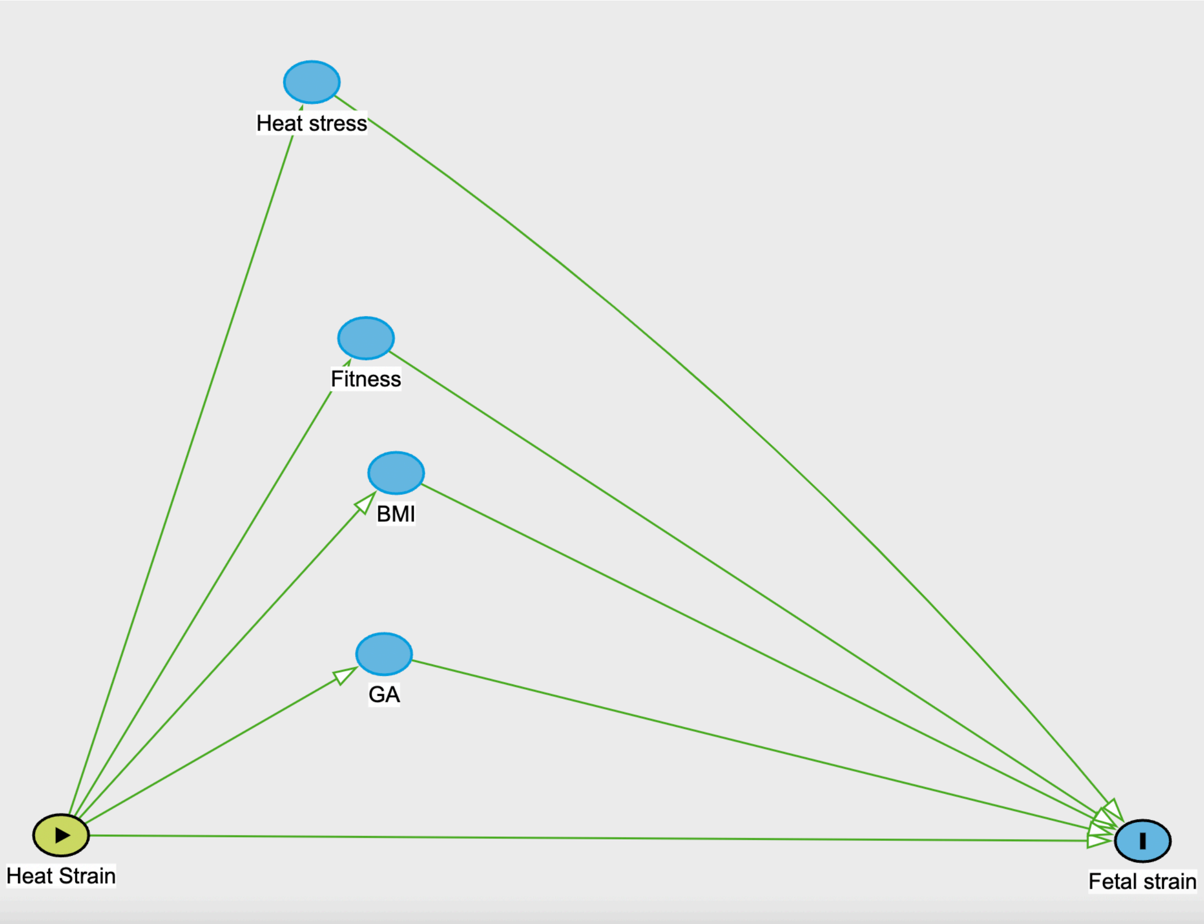

Figure 11: Directed Acyclic Graph of maternal heat strain and fetal strain

R code:
The # on a line indicates notes in the code. Other lines can be run directly in RStudio.

```
#packages
library(dplyr)
library(magrittr)
library(rstatix)
library(ggplot2)
library(ggcorrplot)
library(ggpmisc)
library(ggpubr)
library(RColorBrewer)
library(gridExtra)

#data: t = wide_df gives results for each participant per field visit. a = long_df gives baseline and working
#changes in physiology, environment etc for each participant at each visit.
t <- readRDS("wide_df.rds")
a <- readRDS("long_df.rds")
#table 1 results:

#categorical variables:
dem_soc <- t %>% filter(visit==1)
dem_soc %>% select(dem_ethnicity, soc_occupation, dem_married) %>% summary()


#age - check distribution
```

```
dem_soc %>% shapiro_test(dem_age)
# not normally distributed - present median and IQR

#years in education - check distribution
dem_soc %>% shapiro_test(dem_eduyrs)
# not normally distributed - present median and IQR

dem_soc %>% select(dem_eduyrs) %>% summary()

# obstetric parameter:
gp <- t %>%
  select(dem_screenno, obs_gravida, obs_parity) %>%
  na.omit() %>%
  unique()

gp %>% shapiro_test(obs_gravida)
gp %>% shapiro_test(obs_parity)
#both non parametric - present median and IQR
gp %>% summary()

t %>% shapiro_test(GA_atvisit)
# non parametric - present median and IQR

summary(t$GA_atvisit)

#anthropometric/physical characteristics

t %>% shapiro_test(ant_height)
# normally distributed
summary(t$ant_height)
sd(t$ant_height)

t %>% shapiro_test(ant_weight)
#non-parametric
summary(t$ant_weight)

t %<>% mutate(BMI = (ant_weight/((ant_height/100)^2)))
t %>% shapiro_test(BMI)
summary(t$BMI)

t %<>% mutate(ant_muacave = ((ant_muac1 + ant_muac2)/2))
t %>% shapiro_test(ant_muacave)
#non-parametric

summary(t$ant_muacave)

t %>% shapiro_test(ant_fatpercent)
#non-parametric
summary(t$ant_fatpercent)

# birthoutcomes

table(dem_soc$APO)
table(dem_soc$prem)
table(dem_soc$LBW)
table(dem_soc$sga)
table(dem_soc$alivedead)

#heat illness symptoms
t %<>%
```

```
  mutate(headache = pmax(per_headache_1, per_headache_2, na.rm=T),
         nausea = pmax(per_nausea_1, per_nausea_2, na.rm = T),
         vomiting = pmax(per_vomitting_1, per_vomitting_2, na.rm = T),
         dizziness = pmax(per_dizziness_1, per_dizziness_2, na.rm = T),
         weakness = pmax(per_weakness_1, per_weakness_2, na.rm = T),
         irritability = pmax(per_irritability_1, per_irritability_2, na.rm = T),
         dry_mouth = pmax(per_drymouth_1, per_drymouth_2, na.rm = T),
         muscle_cramp = pmax(per_musclecramps_1, per_musclecramps_2, na.rm = T))

heat_ill <- t %>% select(dem_screenno, visit, headache,
                    nausea, vomiting, dizziness, weakness,
                    irritability, dry_mouth, muscle_cramp)


heat_ill %<>% convert_as_factor(headache,
                        nausea, vomiting, dizziness, weakness,
                        irritability, dry_mouth, muscle_cramp)

# melt data frame to long format
h_melt <- data.table::melt(heat_ill[, c(1, 3:10)], id.vars = "dem_screenno")

h_melt %<>% na.omit()

# add ggplot
Fig5 <- ggplot(data = h_melt) +
  geom_bar(aes(x = variable, fill = value, colour = value), color="black", position=position_dodge()) +
  scale_fill_manual(values=c( "#FFFFFF", "#d8b365")) +
  theme_minimal() +
  ylab("count") +
  xlab("heat illness symptoms") +
  theme(legend.title = element_blank())


table(t$heat_symp)



#### univariable models for supplement table one ####

library(lme4)

a1 <- lmer(psimax ~ fbc_hgb + (1|dem_screenno), data = a)
a2 <- lmer(psimax ~ osmo + (1|dem_screenno), data = a)
a3 <- lmer(psimax ~ fbc_hct + (1|dem_screenno), data = a)
a4 <- lmer(psimax ~ GA_atvisit + (1|dem_screenno), data = a)
a5 <- lmer(psimax ~ tymptem + (1|dem_screenno), data = a)
a6 <- lmer(psimax ~ duration  + (1|dem_screenno), data = a)
a7 <- lmer(psimax ~ MET1_ave + (1|dem_screenno), data = a)
a9 <- lmer(psimax ~ wlk_distance + (1|dem_screenno), data = a)
a10 <- lmer(psimax ~ obs_APO + (1|dem_screenno), data = a)
a11 <- lmer(psimax ~ ant_weight + (1|dem_screenno), data = a)
a12 <- lmer(psimax ~ ant_tbw  + (1|dem_screenno), data = a)
a13 <- lmer(psimax ~ ant_fatmass  + (1|dem_screenno), data = a)
a14 <- lmer(psimax ~ del_sex +  (1|dem_screenno), data = a)
a15 <- lmer(psimax ~ del_weight  + (1|dem_screenno), data = a)
a16 <- lmer(psimax ~ WeightCentile  + (1|dem_screenno), data = a)
a17 <- lmer(psimax ~ GA_days + (1|dem_screenno), data = a)
a18 <- lmer(psimax ~ preg_bp  + (1|dem_screenno), data = a)
a19 <- lmer(psimax ~ dem_age + (1|dem_screenno), data = a)
a20 <- lmer(psimax ~ APO + (1|dem_screenno), data = a)
```

```
a21 <- lmer(psimax ~ preg_inf  + (1|dem_screenno), data = a)
a22 <- lmer(psimax ~ MET1_ave*duration + (1|dem_screenno), data = a)
a23 <- lmer(psimax ~ (1|dem_screenno), data = a)
a24 <- lmer(psimax ~ AE_all + (1|dem_screenno), data = a)
a26 <- lmer(psimax ~ max_wbgt + (1|dem_screenno) , data = a)
a27 <- lmer(psimax ~ ave_wbgt + (1|dem_screenno), data = a)
a28 <- lmer(psimax ~ max_UTCI + (1|dem_screenno), data = a)
a29 <- lmer(psimax ~ ave_UTCI + (1|dem_screenno), data = a)
a30 <- lmer(psimax ~ max_TA + (1|dem_screenno), data = a)
a31 <- lmer(psimax ~ ave_TA + (1|dem_screenno), data = a)
a32 <- lmer(psimax ~ max_RH + (1|dem_screenno), data = a)
a33 <- lmer(psimax ~ ave_RH + (1|dem_screenno), data = a)


#use car::Confint(model) to get estimate and confidence intervals
#use car::Anova(model) to get p values

# univariate analysis of fetal heart rate:

b2 <- lmer(fhr ~ max_UTCI + (1|dem_screenno), data = a)
b3 <- lmer(fhr ~ ave_UTCI + (1|dem_screenno), data = a)
b5 <- lmer(fhr ~ max_wbgt + (1|dem_screenno), data = a)
b6 <- lmer(fhr ~ ave_wbgt + (1|dem_screenno), data = a)
b7 <- lmer(fhr ~ max_TA + (1|dem_screenno), data = a)
b8 <- lmer(fhr ~ ave_TA + (1|dem_screenno), data = a)
b9 <- lmer(fhr ~ max_RH + (1|dem_screenno), data = a)
b10 <- lmer(fhr ~ ave_RH + (1|dem_screenno), data = a)
b11 <- lmer(fhr ~ duration + (1|dem_screenno), data = a)
b12 <- lmer(fhr ~ MET1_ave + (1|dem_screenno), data = a)
b13 <- lmer(fhr ~ AE_all + (1|dem_screenno), data = a)
b14 <- lmer(fhr ~ tymptem + (1|dem_screenno), data = a)
b15 <- lmer(fhr ~ osmo + (1|dem_screenno), data = a)
b16 <- lmer(fhr ~ fbc_hct + (1|dem_screenno), data = a)
b17 <- lmer(fhr ~ ant_tbw + (1|dem_screenno), data = a)
b18 <- lmer(fhr ~ ant_weight + (1|dem_screenno), data = a)
b19 <- lmer(fhr ~ dem_age + (1|dem_screenno), data = a)
b20 <- lmer(fhr ~ wlk_distance + (1|dem_screenno), data = a)
b21 <- lmer(fhr ~ fbc_hgb + (1|dem_screenno), data = a)
b22 <- lmer(fhr ~ ant_fatmass + (1|dem_screenno), data = a)
b23 <- lmer(fhr ~ GA_atvisit + (1|dem_screenno), data = a)
b24 <- lmer(fhr ~ obs_APO + (1|dem_screenno), data = a)
b25 <- lmer(fhr ~ APO + (1|dem_screenno), data = a)
b26 <- lmer(fhr ~ skin_temp + (1|dem_screenno), data = a)
b27 <- lmer(fhr ~ heartrate + (1|dem_screenno), data = a)
b28 <- lmer(fhr ~ psimax + (1|dem_screenno), data = a)

#use car::Confint(model) to get estimate and confidence intervals
#use car::Anova(model) to get p values


# fetal stress as a categorical outcome

a$fetal_stress <- as.factor(a$fetal_stress)

c2 <- glmer(fetal_stress ~ max_UTCI + (1|dem_screenno), data = a, family = binomial)
c3 <- glmer(fetal_stress ~ ave_UTCI + (1|dem_screenno), data = a, family = binomial)
c5 <- glmer(fetal_stress ~ max_wbgt + (1|dem_screenno), data = a, family = binomial)
c6 <- glmer(fetal_stress ~ ave_wbgt + (1|dem_screenno), data = a, family = binomial)
c7 <- glmer(fetal_stress ~ max_TA + (1|dem_screenno), data = a, family = binomial)
c8 <- glmer(fetal_stress ~ ave_TA + (1|dem_screenno), data = a, family = binomial)
```

```
c9 <- glmer(fetal_stress ~ max_RH + (1|dem_screenno), data = a, family = binomial)
c10 <- glmer(fetal_stress ~ ave_RH + (1|dem_screenno), data = a, family = binomial)
c11 <- glmer(fetal_stress ~ duration + (1|dem_screenno), data = a, family = binomial)
c12 <- glmer(fetal_stress ~ MET1_ave + (1|dem_screenno), data = a, family = binomial)
c13 <- glmer(fetal_stress ~ AE_all + (1|dem_screenno), data = a, family = binomial)
c14 <- glmer(fetal_stress ~ tymptem + (1|dem_screenno), data = a, family = binomial)
c15 <- glmer(fetal_stress ~ osmo + (1|dem_screenno), data = a, family = binomial)
c16 <- glmer(fetal_stress ~ fbc_hct + (1|dem_screenno), data = a, family = binomial)
c17 <- glmer(fetal_stress ~ ant_tbw + (1|dem_screenno), data = a, family = binomial)
c18 <- glmer(fetal_stress ~ ant_weight + (1|dem_screenno), data = a, family = binomial)
c19 <- glmer(fetal_stress ~ dem_age + (1|dem_screenno), data = a, family = binomial)
c20 <- glmer(fetal_stress ~ wlk_distance + (1|dem_screenno), data = a, family = binomial)
c21 <- glmer(fetal_stress ~ fbc_hgb + (1|dem_screenno), data = a, family = binomial)
c22 <- glmer(fetal_stress ~ ant_fatmass + (1|dem_screenno), data = a, family = binomial)
c23 <- glmer(fetal_stress ~ GA_atvisit + (1|dem_screenno), data = a, family = binomial)
c24 <- glmer(fetal_stress ~ obs_APO + (1|dem_screenno), data = a, family = binomial)
c25 <- glmer(fetal_stress ~ APO + (1|dem_screenno), data = a, family = binomial)
c26 <- glmer(fetal_stress ~ skin_temp + (1|dem_screenno), data = a, family = binomial)
c27 <- glmer(fetal_stress ~ heartrate + (1|dem_screenno), data = a, family = binomial)
c28 <- glmer(fetal_stress ~ psimax + (1|dem_screenno), data = a, family = binomial)


## to get odds ratios

fs_list <- list(c2,c3,c5,c6,c7,c8,c9,c10,c11,c12,c13,
c14,c15,c16,c17,c18,c19,c20,c21,c22,c23,c24,
c25, c26,c27, c28)

### write functions
get_se <- function(x){sqrt(diag(vcov(x)))}
get_tab <- function(x,y){
  cbind(Est = fixef(x), LL = fixef(x) - 1.96 * y, UL = fixef(x) + 1.96 *y)
}

get_or <- function(x){exp(x)}

### list of OR with CI.

se_list <- map(fs_list, get_se)
tab_list <- map2(fs_list, se_list, get_tab)
or_list <- map(tab_list, get_or)
or_list


## to get p value
pv_list <- map(fs_list, car::Anova)


#### heat stress and heat strain ####

#remove resting state
ab <- a %>% filter(psi > 0)

locknots <- quantile(ab$max_wbgt,c(0.25, 0.9), na.rm=T) # location of the knots of the spline (in the median
and 90th percentile of the tmean distribution)
locknot2 <- quantile(ab$max_wbgt, c(0.5), na.rm = T)
locknot3 <- quantile(ab$max_wbgt, c(0.5, 0.9), na.rm=T)

wbgtmaxb1 <- onebasis(ab$max_wbgt, fun="lin")
wbgtmaxb2 <- onebasis(ab$max_wbgt, fun="ns", knots = locknot2)
```

```
wbgtmaxb3 <- onebasis(ab$max_wbgt, fun="bs", degree=2, knots=locknots)
wbgtmaxb4 <- onebasis(ab$max_wbgt, fun="bs", degree=2, knots=locknot2)
wbgtmaxb5 <- onebasis(ab$max_wbgt, fun="bs", degree=2, knots=locknot3)

# RUN MODELS
psi1 <- lm(psimax ~ wbgtmaxb1, ab)  # LINEAR
psi2 <- lm(psimax ~ wbgtmaxb2, ab)  # NON-LINEAR WITH NS
psi3 <- lm(psimax ~ wbgtmaxb3, ab)  # NON-LINEAR WITH BS
psi4 <- lm(psimax ~ wbgtmaxb4, ab)
psi5 <- lm(psimax ~ wbgtmaxb5, ab)

AIC(psi1)
AIC(psi2)
AIC(psi3)
AIC(psi4)
AIC(psi5)

# PREDICT
predpsi1 <- crosspred(wbgtmaxb1,psi1)
plot(predpsi1)

# UTCI
ac <- ab %>% filter(max_UTCI <45)

nots <- quantile(ac$max_UTCI,c(0.25, 0.9), na.rm=T) # location of the knots of the spline (in the median and 90th percentile of the tmean distribution)
not2 <- quantile(ac$max_UTCI, c(0.5), na.rm = T)
not3 <- quantile(ac$max_UTCI, c(0.5, 0.9), na.rm=T)


utcimaxb1 <- onebasis(ac$max_UTCI, fun="lin")
utcimaxb2 <- onebasis(ac$max_UTCI, fun="ns", knots = not2)
utcimaxb3 <- onebasis(ac$max_UTCI, fun="bs", degree=2, knots=nots)
utcimaxb4 <- onebasis(ac$max_UTCI, fun="bs", degree=2, knots=not2)
utcimaxb5 <- onebasis(ac$max_UTCI, fun="bs", degree=2, knots=not3)


# RUN MODELS
psia <- lm(psimax ~ utcimaxb1, ac)  # LINEAR
psib <- lm(psimax ~ utcimaxb2, ac)  # NON-LINEAR WITH NS
psic <- lm(psimax ~ utcimaxb3, ac)  # NON-LINEAR WITH BS
psid <- lm(psimax ~ utcimaxb4, ac)
psie <- lm(psimax ~ utcimaxb5, ac)

AIC(psia)
AIC(psib)
AIC(psic)
AIC(psid)
AIC(psie)

predpsia <- crosspred(utcimaxb1, psia)
plot(predpsia)



#### Multivariable models ####

# functions
get_se <- function(x){sqrt(diag(vcov(x)))}
get_tab <- function(x,y){
```

```
  cbind(Est = fixef(x), LL = fixef(x) - 1.96 * y, UL = fixef(x) + 1.96 *y)
}

get_or <- function(x){exp(x)}

#### fetal strain ###
## Model 1

mod_1 <- glmer(fetal_stress ~ wbgtmaxb1 + (1|dem_screenno), data = a, family = binomial)
mod_2 <- glmer(fetal_stress ~ utcimaxb1 + (1|dem_screenno), data = a, family = binomial)

se_mod1 <- get_se(mod_1)
tab_mod1 <- get_tab(mod_1, se_mod1)
or_tab_mod1 <- get_or(tab_mod1)
or_tab_mod1

se_mod2 <- get_se(mod_2)
tab_mod2 <- get_tab(mod_2, se_mod2)
or_tab_mod2 <- get_or(tab_mod2)
or_tab_mod2


## model 2 ## heat stress and heat strain to get adjusted direct effects based
# on a priori assumptions and DAG

mod_3 <- glmer(fetal_stress ~ wbgtmaxb1 + psimax + (1|dem_screenno), data = a, family = binomial)

mod_4 <- glmer(fetal_stress ~ utcimaxb1 + psimax + (1|dem_screenno), data = a, family = binomial)

summary(mod_3)
summary(mod_4)

se_mod3 <- get_se(mod_3)
tab_mod3 <- get_tab(mod_3, se_mod3)
or_tab_mod3 <- get_or(tab_mod3)
or_tab_mod3

se_mod4 <- get_se(mod_4)
tab_mod4 <- get_tab(mod_4, se_mod4)
or_tab_mod4 <- get_or(tab_mod4)
or_tab_mod4

## determine heat strain and fetal strain adjusted for confounders

mod_5 <- glmer(fetal_stress ~ wbgtmaxb1 + psimax + GA_atvisit + wlk_distance
          + ant_fatmass + (1|dem_screenno), data = a, family = binomial)

mod_6 <- glmer(fetal_stress ~ utcimaxb1 + psimax + GA_atvisit + wlk_distance
          + ant_fatmass + (1|dem_screenno), data = a, family = binomial)

summary(mod_5)
summary(mod_6)

se_mod5 <- get_se(mod_5)
tab_mod5 <- get_tab(mod_5, se_mod5)
or_tab_mod5 <- get_or(tab_mod5)
or_tab_mod5

se_mod6 <- get_se(mod_6)
tab_mod6 <- get_tab(mod_6, se_mod6)
```

```
or_tab_mod6 <- get_or(tab_mod6)
or_tab_mod6


#### same but for fetal heart rate

## model 1 ## total effect of heat stress of FHR

mod_7 <- lmer(fhr ~ utcimaxb1 + (1|dem_screenno), data = a)

summary(mod_7)
AIC(mod_7)
car::Anova(mod_7)
confint(mod_7)


mod_8 <- lmer(fhr ~ wbgtmaxb1 + (1|dem_screenno), data = a)

summary(mod_8)
AIC(mod_8)
car::Anova(mod_8)
confint(mod_8)

pred_8 <- crosspred(wbgtmaxb1, mod_8)
pred_7 <- crosspred(utcimaxb1, mod_7)

## model 2

mod_9 <- lmer(fhr ~ utcimaxb1 + psimax + (1|dem_screenno), data = a)

summary(mod_9)
AIC(mod_9)
car::Anova(mod_9)
confint(mod_9)

mod_10 <- lmer(fhr ~ wbgtmaxb1 + psimax + (1|dem_screenno), data = a)

summary(mod_10)
AIC(mod_10)
car::Anova(mod_10)
confint(mod_10)

## model 3

mod_11 <- lmer(fhr ~ utcimaxb1 + psimax+ ant_fatmass + GA_atvisit
        + wlk_distance + (1|dem_screenno), data = a)

summary(mod_11)
AIC(mod_11)
car::Anova(mod_11)
confint(mod_11)

mod_12 <- lmer(fhr ~ wbgtmaxb1 + psimax+ ant_fatmass + GA_atvisit
         + wlk_distance + (1|dem_screenno), data = a)

summary(mod_12)
AIC(mod_12)
car::Anova(mod_12)
confint(mod_12)
```

```
#test model assumptions:
ab <- a %>% select(dem_screenno, wlk_distance, GA_atvisit, ant_fatmass, psimax, max_wbgt)
Plot.Model <-plot(resid(mod_12),ab$fhr)

#test homogeneity of variance using Levene test:

ab$Model.fhr.Res<- residuals(mod_12) #extracts the residuals and places them in a new column in our original
data table
ab$Abs.fhr.Res <-abs(ab$Model.fhr.Res) #creates a new column with the absolute value of the residuals
ab$Model.fhr.Res2 <- ab$Abs.fhr.Res^2 #squares the absolute values of the residuals to provide the more
robust estimate
Levene.Model.fhr <- lm(Model.fhr.Res2 ~ dem_screenno, data=ab) #ANOVA of the squared residuals
anova(Levene.Model.fhr) #displays the results


#### visualisations ####

##Fig 2

t$wbgt_cat2 <- factor(t$wbgt_cat, levels = rev(levels(t$wbgt_cat)))
t$UTCI_cat2 <- factor(t$UTCI_cat, levels = rev(levels(t$UTCI_cat)))
t1 <- t %>% select(wlk_date, max_UTCI, UTCI_cat2)
t1 %<>% na.omit()

b4 <- ggplot(t,aes(wlk_date, max_wbgt)) +
  geom_point(aes(colour = factor(wbgt_cat2)), size =2)+
   labs(title = "Wet Bulb Globe Temperature", x = "month", y = "WBGT(\u00B0C)") +
   scale_colour_brewer(palette = "RdYlGn", name = "WBGT category") +
   theme_bw()
 b4
>b5 <- ggplot(t1,aes(wlk_date, max_UTCI)) +
 geom_point(aes(colour = factor(UTCI_cat2)), size =2) +
  labs(title = "Universal Thermal Comfort Index", x = "month", y = "UTCI(\u00B0C)") +
   scale_color_brewer(palette = "RdYlGn", name = "UTCI category", labels = c("EH", "VSH", "SH", "MH")) +
   theme_bw()
 b5

 Fig2 <- grid.arrange(b4, b5, ncol=2)

## Fig 3

#gather for change in temp

mat_temp <- t %>% select(dem_screenno, TBS, Tmax)

mat <- mat_temp %>% tidyr::pivot_longer(c(TBS, Tmax), names_to = "temperature", values_to = "degree")

#change in skin temperature:

skt_temp <- t %>% select(dem_screenno, cam_chesttemp_1, SKT.Max.)
skt_temp %<>% na.omit()

skt <- skt_temp %>% tidyr::pivot_longer(c(cam_chesttemp_1, SKT.Max.), names_to = "temperature",
values_to = "degree")

b11 <- ggplot(mat, aes(x = temperature, y = degree, group = temperature, fill = temperature)) +
 geom_boxplot(outlier.shape = 1) +
 labs(title = "Change in mean maternal temperature", x = "Time of day", y = "Tympanic temperature
(\u00B0C)") +
```

```
 scale_fill_brewer(palette = "Dark2", name = "Time of day", labels = c("Baseline", "Working")) +
 theme_bw() +
   theme(axis.text.x = element_blank()) +
 stat_compare_means(paired = T, label.x = 1, label.y = 38.6)

 b11

 b11b <- ggplot(skt, aes(x = temperature, y = degree, group = temperature, fill = temperature)) +
   geom_boxplot(outlier.shape = 1) +
   labs(title = "Change in mean maternal skin temperature", x = "Time of day", y = "Skin temperature
(\u00B0C)") +
   scale_fill_brewer(palette = "Dark2", name = "Time of day", labels = c("Baseline", "Working")) +
   theme_bw() +
   theme(axis.text.x = element_blank()) +
   stat_compare_means(paired = T, label.x = 1, label.y = 41.1)

 b11b

Fig3 <- grid.arrange(b11, b11b, ncol=2)


## fig4 – output from multilevel model

par(mfrow=c(1,2))
plot(pred_8, xlab = expression(paste("WBGT (",degree,"C)")),
     ylab = "Change in FHR",
     col = "dark red")
title(main = "Wet Bulb Globe Temperature")
plot(pred_7, xlab = expression(paste("UTCI (",degree,"C)")),
     ylab = "Change in FHR",
     col = "dark red")
title(main = "Universal Thermal Climate Index")
```
